# Temporal sequences of brain activity at rest are constrained by white matter structure and modulated by cognitive demands

**DOI:** 10.1038/s42003-020-0961-x

**Published:** 2020-05-22

**Authors:** Eli J. Cornblath, Arian Ashourvan, Jason Z. Kim, Richard F. Betzel, Rastko Ciric, Azeez Adebimpe, Graham L. Baum, Xiaosong He, Kosha Ruparel, Tyler M. Moore, Ruben C. Gur, Raquel E. Gur, Russell T. Shinohara, David R. Roalf, Theodore D. Satterthwaite, Danielle S. Bassett

**Affiliations:** 1 Department of Neuroscience, Perelman School of Medicine, Philadelphia, PA 19104 USA; 2 Department of Bioengineering, School of Engineering & Applied Science, Philadelphia, PA 19104 USA; 3 Department of Psychiatry, Perelman School of Medicine, Philadelphia, PA 19104 USA; 4 Department of Biostatistics, Epidemiology, & Informatics, Perelman School of Medicine, Philadelphia, PA 19104 USA; 5 Department of Physics & Astronomy, College of Arts & Sciences, Philadelphia, PA 19104 USA; 6 Department of Neurology, Perelman School of Medicine, Philadelphia, PA 19104 USA; 7 Department of Electrical & Systems Engineering, School of Engineering & Applied Science, Philadelphia, PA 19104 USA; 8 Department of Radiology, Perelman School of Medicine, University of Pennsylvania, Philadelphia, PA 19104 USA; 9 Santa Fe Institute, Santa Fe, NM 87501 USA

**Keywords:** Network models, Working memory

## Abstract

A diverse set of white matter connections supports seamless transitions between cognitive states. However, it remains unclear how these connections guide the temporal progression of large-scale brain activity patterns in different cognitive states. Here, we analyze the brain’s trajectories across a set of single time point activity patterns from functional magnetic resonance imaging data acquired during the resting state and an n-back working memory task. We find that specific temporal sequences of brain activity are modulated by cognitive load, associated with age, and related to task performance. Using diffusion-weighted imaging acquired from the same subjects, we apply tools from network control theory to show that linear spread of activity along white matter connections constrains the probabilities of these sequences at rest, while stimulus-driven visual inputs explain the sequences observed during the n-back task. Overall, these results elucidate the structural underpinnings of cognitively and developmentally relevant spatiotemporal brain dynamics.

## Abstract (web summary)

Eli J. Cornblath et al use tools from linear network control theory to show that white matter connectivity constrains transitions between brain activity patterns at rest to favor transitions with small energy requirements, while visual inputs overcome these constraints during a cognitive task. These findings highlight the importance of accounting for both internal white matter network dynamics and external inputs in models of brain activity.

## Introduction

An elusive goal of computational neuroscience is to describe the brain as a dynamical system with a predictable natural temporal evolution and response to input. Such a model would be invaluable to clinicians as a generalizable tool for identifying optimal brain stimulation approaches to drive the brain from various states of disease to states of health^1^. Yet, the endeavor of identifying a real non-linear dynamical system that provides such insights is exceedingly difficult, in part due to the high dimensionality of brain activity and the complex nature of the brain’s intrinsic functional interactions. It is known that the white matter architecture of the brain contributes to the diverse patterns of activity and functional connectivity that represent information processing underlying cognitive function^2–5^. However, the exact manner in which white matter connectivity constrains the temporal dynamics of brain activity remains poorly understood. Improving our understanding requires a rich characterization of time-varying brain activity, as well as a robust model to link brain structure with brain activity.

**Fig. 1:**
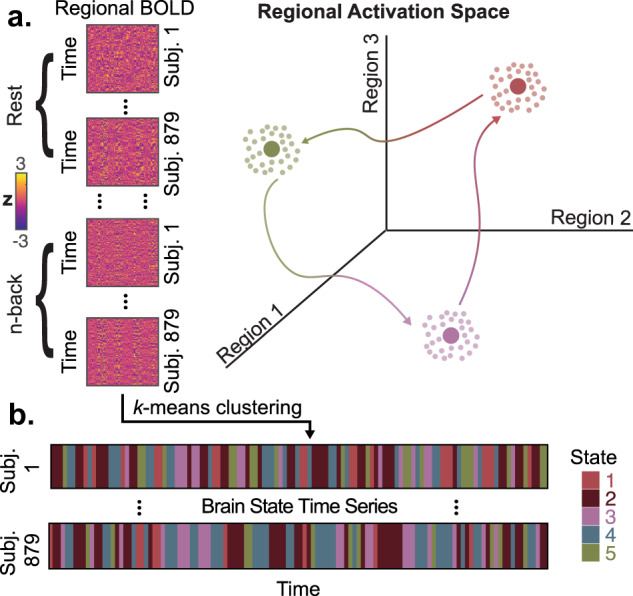
**Schematic of methods for functional image analysis**. **a** Regional BOLD time series from resting-state and n-back task scans are concatenated across subjects. Each row in this concatenated data matrix represents a point in a high-dimensional space whose axes correspond to regional activity. A schematic of a low-dimensional version of this space is shown as an example on the right. Our goal is to identify frequently visited locations in this space and study the temporal progression between these locations during rest and task. **b** We then apply a *k*-means clustering algorithm to generate a series of cluster labels that can be mapped back to individual subjects, producing subject-specific brain state time series.

Myriad approaches have been applied to resting functional magnetic resonance imaging (fMRI) to understand intrinsic brain dynamics. The most common approach (“functional connectivity”) involves analyzing the correlations between the activity time series of pairs of brain regions. While pairwise correlation-based approaches summarize inter-regional synchrony over a period of time, cutting-edge signal-processing approaches to fMRI can provide a richer account of brain dynamics by considering the whole-brain patterns of activity at single time points^6–13^. One can conceive of the brain as progressing through a state space whose axes correspond to the activity at each region^4,14^ (Fig. 1a). Each point in this space corresponds to an observed pattern of brain activity, and the sequential trajectories through this space represent how brain activity patterns change over time. This approach allows one to utilize the maximum temporal resolution offered by BOLD fMRI, unlike many dynamic functional connectivity methods, which are limited by a minimum window size^15^. Studies analyzing the brain’s regional activation space have found that frequently visited activity patterns consist of different combinations of RSN components^6,8,9,16,17^. Brain activity patterns are known to represent information content^18^, distinct modes of information processing^19,20^, and attention to stimuli^20,21^. Such activation patterns occur both at rest and in the presence of tasks or attentional demands, and are often considered to be neural representations of cognitive state^4,14,22^.

However, a fundamental understanding of the brain’s trajectories through regional activation space has been limited by the use of thresholding that disrupts the continuity of the time series^6–8^, a focus on between-scan rather than within-scan differences^14^, a narrow focus on only a few brain regions^10^, and various modeling assumptions impacting the nature of the temporal dynamics detected^9^. Such limitations have also hampered progress in understanding how state-space trajectories might be constrained by or indeed supported by underlying brain structure. One intriguing possibility is that the white matter architecture of the brain is designed to support coordinated activity within RSNs and information transfer between RSNs, which might be reflected in the temporal progression between distinct states of RSN coactivation. For instance, one could imagine that coactivation of visual regions with dorsal attention regions, followed by activation of frontoparietal executive control regions might reflect reception, integration, and higher order processing of a visual stimulus. Critically, the normative neurodevelopment of time-resolved brain state dynamics and their cognitive relevance also remain unknown, limiting our ability to incorporate such neurobiological features into our understanding of neuropsychiatric disorders with developmental origins^23–26^. Specific neuropsychiatric symptoms, such as hallucinations or negative rumination, may be represented in coactivation patterns and their temporal dynamics, which could be disrupted with brain stimulation^27–30^.

To address these fundamental gaps in knowledge, we consider a large, community-based sample (*n* = 879) of healthy youth from the Philadelphia Neurodevelopmental Cohort (PNC)^25,31^, all of whom underwent diffusion-weighted and T1-weighted structural imaging, passive fixation resting state fMRI, and n-back working memory task fMRI^32–34^. We begin by using *k*-means clustering to extract a set of discrete brain states from the fMRI data^7,8,11,35^, and to assign each functional volume from both rest and task scans to one of those states. We hypothesize that the brain’s temporal progression between different states is influenced by cognitive demands and stimuli, which we test by quantifying the time that subjects dwell within states, and the propensity to transition between states. Next, we hypothesize that structural connectivity constrains the temporal progression of brain states and explains why these particular brain states exist. We test these hypotheses using emerging tools from network control theory^27,36–39^, along with comparison to stringent null models^40,41^ to ensure the specificity of our findings. Finally, we hypothesize that brain state dynamics change throughout development to optimize cognitive performance.

By rigorously testing these hypotheses, we find increased temporal persistence of a state associated with high activity in frontoparietal cortex during task. On the other hand, states associated with coherent activity in default mode areas have similar temporal persistence between rest and task with an increased rate of appearance during rest. Interestingly, two divergent trajectories towards frontoparietal and default mode states following from a sensory-driven state are positively and negatively related to task performance, respectively. Using tools from linear network control theory, we show that state transitions with small energy requirements given the brain’s white matter architecture occur more frequently in the observed data than state transitions with large energy requirements. Additionally, accounting for visual input explains the differences in state-space trajectories between rest and task. Finally, we show that brain state dynamics and predicted energies of state transitions are associated with age and explain individual differences in working memory performance. Overall, we demonstrate the utility of state-space models in understanding the structural basis for developmentally and cognitively relevant context-dependent brain dynamics.

## Results

### Brain states capture instantaneous coactivation between resting state functional networks

The spatiotemporal dynamics of brain activity are exceedingly complex and not fully understood. Analyzing pairwise correlations between regions over time ("functional connectivity" or FC) is a common approach used to quantify interactions between brain regions. However, static FC does not necessarily account for spontaneous or stimulus-evoked coactivation observed at single time frames (Supplementary Fig. 1), which is the maximum temporal resolution offered by BOLD fMRI for a given repetition time (TR)^8,42^. Here, we used *k*-means clustering^3,8,35^ to assign each time point from resting and n-back task fMRI scans into clusters of statistically similar and temporally recurrent whole-brain spatial coactivation patterns, hereafter referred to as “brain states” (Fig. 1a). We found that little additional variance in BOLD signal was explained by increasing *k* beyond 5, and the clustering solution at *k* = 5 demonstrated high reliability in split-half resampling (Supplementary Fig. 2). Importantly, we found that these BOLD data exhibited clustering in regional activation space beyond what would be expected from signals with the same autocorrelation profiles (Supplementary Fig. 3a). Additionally, states were highly similar between rest and n-back task scans (Supplementary Fig. 4b), in a second parcellation (Supplementary Fig. 5a), in an independent sample with older subjects (Supplementary Fig. 6a–c), and after removal of high-motion frames (Supplementary Fig. 7a, b).

**Fig. 2:**
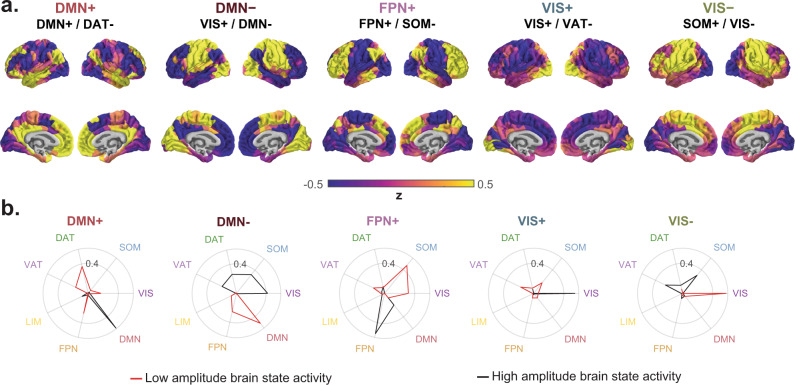
**Brain states represent coactivation within and between resting state functional networks**. **a** Brain states defined as the centroids of clusters identified using an unsupervised machine-learning algorithm applied to rest and n-back task fMRI data. Brain states are labeled based on cosine similarity with a priori resting state functional networks (RSNs)^43^. The top label corresponds to the RSN with the most overall similarity, and the bottom two labels separated by a forward slash reflect the RSNs with the most similarity to the positive and negative components of each state, respectively. **b** Cosine similarity between positive (black) and negative (red) components of each state with binary state vectors corresponding to a priori definitions of RSNs^43^. Larger radial values correspond to higher cosine similarity. DAT dorsal attention network, DMN default mode network, FPN frontoparietal network, LIM limbic network, SOM somatomotor network, VAT ventral attention network, and VIS visual network.

We found that “resting-state functional networks” (RSNs)^43,44^, groups of regions with stronger static FC with each other than with other regions, exhibited coherent high or low amplitude activity within each cluster centroid. This finding is consistent with strong within-network FC. Due to their similarity to RSNs, we named each of the five states that we observed after the previous RSN whose isolated high or low amplitude activity best explained each state. This choice did not influence any analyses and is solely for convenient interpretation. We refer to them as the DMN+, DMN−, FPN+, VIS+, and VIS−, representing activity above (+) or below (−) regional means in default mode (DMN), frontoparietal (FPN), and visual networks (VIS), respectively (Fig. 2a). We also asked which additional RSNs exhibited coherent activity in each state by quantifying the alignment of the high and low amplitude components of each brain state activity pattern separately with each RSN, indicating the presence of coherent activity within somatomotor network (SOM), dorsal attention network (DAT), and ventral attention network (VAT) (Fig. 2b).

Interestingly, in addition to coherent activity within each RSN, we found that centroids contained multiple RSNs simultaneously exhibiting coherent high or low amplitude activity. For example, the DMN exhibited high amplitude while the DAT simultaneously exhibited low amplitude in the DMN+ state. This spatial organization likely reflects known patterns of between-network FC between task-positive and task-negative systems^45^ (Supplementary Fig. 8a, mean *r* = −0.10, one-sample *t*-test, df = 878, *t* = −80.45, *p* < 10^−15^). However, the DMN−, VIS+, and VIS− states evidence unexpected, transient patterns of coactivation between VIS and SOM systems. Specifically, in the DMN− state, SOM and VIS regions were both at high amplitude (Fig. 2b). In the VIS+ state, SOM regions were at low amplitude and VIS regions were at high amplitude (Fig. 2b). In the VIS− state, SOM regions were at high amplitude and VIS regions were at low amplitude (Fig. 2b). Despite the presence of three unique coactivation patterns between these two RSNs, the mean FC between regions in VIS and SOM did not significantly differ from 0 (Supplementary Fig. 8a, mean *r* = −0.0012, one-sample *t*-test, df = 878, *t* = −0.085, *p* = 0.40). These patterns of simultaneous activation and deactivation provide a snapshot of instantaneous interactions between RSNs that could not be obtained through the analysis of FC.

### Temporal patterns of brain state occurrence and occupancy

**Fig. 3:**
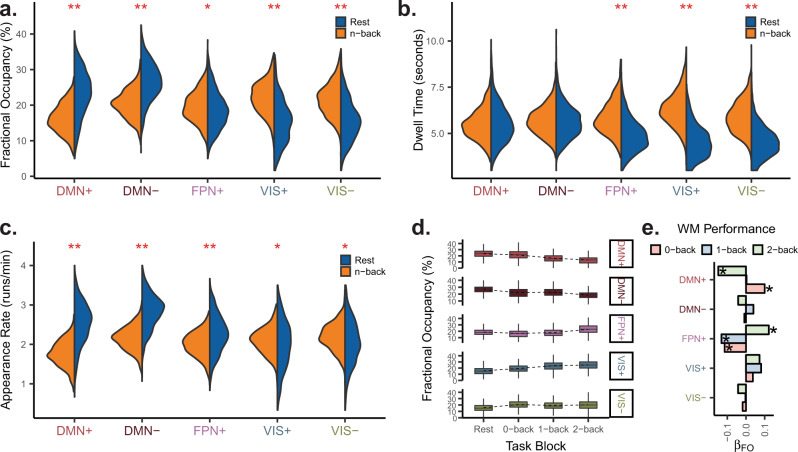
**Brain state occupancy and temporal persistence are modulated by task demands**. **a**–**c** Distributions of subject-level fractional occupancy (panel **a**), dwell time (panel **b**), and appearance rate (panel **c**), for each brain state in rest and task. DMN states exhibit higher fractional occupancies and appearance rates during rest and VIS states exhibit higher fractional occupancies and dwell times during task. **p_corr_ < 10^−15^, *p_corr_ < 10^−4^, paired *t*-tests Bonferroni-corrected across *k* = 5 states separately for fractional occupancy, dwell time, and run rate. **d** Within task scans, the state fractional occupancies change with increasing cognitive load. The dashed lines indicate the mean across subjects. Boxplot rectangles show the median, 25th, and 75th percentiles of fractional occupancy for each block, and whiskers show 1.5 times the interquartile range. **e** Standardized linear regression *β* weights for state-specific fractional occupancy (FO) on working memory (WM) performance for each task block requiring an increasing WM load (0-back, 1-back, and 2-back). We found opposing trends for DMN+ and FPN+ states from 0-back to 2-back. WM working memory, FO fractional occupancy.

After identifying large-scale brain states representing instantaneous coactivation between RSNs, we were interested in comparing the dynamics of brain state occupancy and dwelling between rest and n-back scans (Fig. 3a). To provide a rich characterization of the dynamics of brain state occupancy, we defined and studied three related metrics for each state: (1) fractional occupancy, the percentage of frames assigned to a state for a given scan or condition, (2) dwell time, the mean duration in seconds of temporally continuous runs of state occupancy, and (3) appearance rate, the number of times a run of any length appeared per minute. Using paired *t*-tests, we assessed whether the population means of subject-specific differences between n-back and rest (*μ*_nback−rest_) for each of these metrics were different from 0. Here, we focus on the FPN and DMN, whose activation and suppression, respectively, are classically seen during cognitively demanding tasks^45–47^.

During the n-back task, we observed lower fractional occupancies in the two default mode states (paired *t*-tests, df = 878, *t* = −31.38, DMN+: *μ*_nback−rest_ = −7.10, *p*_corr_ < 10^−15^, DMN−: *μ*_nback−rest_ = −6.15, df = 878, *t* = 30.57, *p*_corr_ < 10^−15^). However, higher fractional occupancy in DMN states at rest was best explained by increased appearance probability of DMN states at rest (paired *t*-tests, DMN+: *μ*_nback−rest_ = −0.77, df = 878, *t* = −40.03, *p*_corr_ < 10^−15^, DMN−: *μ*_nback−rest_ = −0.68, df = 878, *t* = −40.74, *p*_corr_ < 10^−15^), while dwell time in DMN states did not differ between rest and task (paired *t*-tests, DMN+: *μ*_nback−rest_ = −0.01, df = 878, *t* = −0.17, *p*_corr_ = 1, DMN−: *μ*_nback−rest_ = 0.06, df = 878, *t* = 1.45, *p*_corr_ = 0.73). Lower DMN+ state fractional occupancies during the n-back task is consistent with DMN suppression observed during attention-demanding tasks^46^. However, the high DMN− fractional occupancy suggests that coherent DMN suppression is not specific to task conditions, and may occur in the context of a unique, transient interaction with primary sensory areas (Fig. 3a). Interestingly, FPN+ state fractional occupancy was similar between rest and task, despite higher dwell time with a lower appearance rate in the n-back task (paired *t*-tests, FPN+ dwell time: *μ*_nback−rest_ = 0.95, df = 878, *t* = 23.69, *p*_corr_ < 10^−15^, FPN+ appearance rate: *μ*_nback−rest_ = −0.26, df = 878, *t* = −14.74, *p*_corr_ < 10^−15^). These findings suggest that the FPN is activated more frequently, albeit transiently, at rest, while sustained activation of the FPN is found during the n-back working memory task.

Next, we decided to examine the dynamics of DMN suppression and FPN activation as a function of cognitive load within the n-back task and as a predictor of task performance. We hypothesized that as cognitive load increased, DMN+ fractional occupancy would decrease and FPN+ fractional occupancy would increase. As expected, the FPN state fractional occupancy increased from the 0-back to the 2-back block (Fig. 3d). Interestingly, spatially anticorrelated DMN states both decreased with increasing cognitive load (Fig. 3d). This finding suggests that working memory involves reduced representation of brain states with coherent activity in the DMN, whether high or low amplitude, and increased representation of the high amplitude FPN state, clarifying the roles of task-positive and task-negative networks^45–47^. Next, when we examined associations between fractional occupancy and block-specific working memory performance (Fig. 2c and d), we found that increasing FPN+ fractional occupancy (Fig. 3e; multiple linear regression, standardized *β*_FO_ = 0.12, df = 872, *t* = 3.85, *p*_corr_ = 1.9 × 10^−3^) and decreasing DMN+ fractional occupancy (Fig. 3e; multiple linear regression, standardized *β*_FO_ = −0.15, df = 872, *t* = −4.71, *p*_corr_ = 4.4 × 10^−5^) were associated with working memory performance during the 2-back block. However, for 0-back blocks, these trends were reversed (Fig. 3e, multiple linear regression; 0-back FPN+, standardized *β*_FO_ = −0.11, df = 872, *t* = −3.49, *p*_corr_ = 7.7 × 10^−3^; 0-back DMN+, standardized *β*_FO_ = 0.10, df = 872, *t* = 2.96, *p*_corr_ = 0.047). This pattern of results might reflect the engagement of alternative systems for low difficulty tasks by strong performers, thus introducing a layer of complexity to the notion of DMN and FPN as primary task-negative and task-positive systems^45^.

### Transitions between brain states

After demonstrating that cognitive demands influence dwell times *in* large-scale brain states, we were interested in how cognitive demands would affect transitions between large-scale brain states. We conceptualized brain state transitions as directional trajectories between different locations in a high-dimensional space whose axes correspond to the level of activity in each brain region. Neuroimaging studies suggest that the brain progresses along a low-dimensional manifold in regional activation space^4,14^, but it remains unknown the extent to which specific trajectories in this space are influenced by cognitive demands and may represent cognitive processes.

**Fig. 4:**
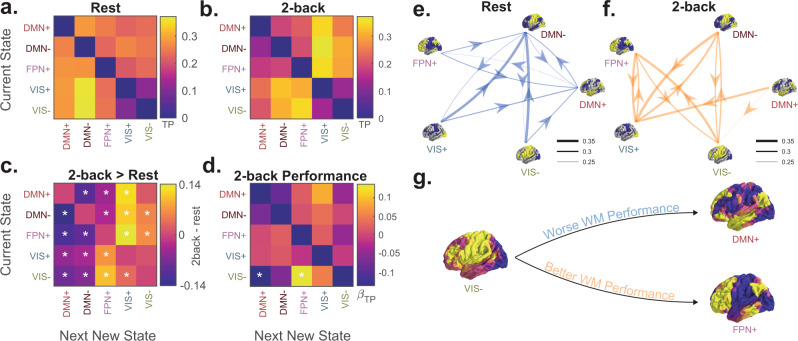
**Brain state transitions are influenced by task demands and related to behavior**. **a** and **b** Group average state transition probability matrices for resting state scans and the 2-back condition. Matrix elements reflect the probability of a state transition after removing the effects of state autocorrelation. **c** Non-parametric permutation testing demonstrating differences between the rest and n-back group average transition probability matrices. *p_corr_ < 0.05, after Bonferroni correction over 20 transition probabilities. **d** Standardized linear regression *β* weights for the transition probability during the 2-back condition of the n-back task as a predictor of task performance during the 2-back condition. Transitions from the VIS− state into the DMN+ and FPN+ states are negatively and positively associated with better performance, respectively. *p_corr_ < 0.05, after Bonferroni correction over 20 transition probabilities. TP transition probability. **e** and **f** Graphical representation of resting state (panel **e**) and 2-back (panel **f**) transition probability matrices as networks whose nodes are states, and whose edges are transition probabilities thresholded at 0.25. **g** Graphical representation of results shown in panel **d**.

Here, in order to study the relationship between cognition and progression through regional activation space, we computed transition matrices for each subject’s resting state scan, n-back task scan, and each condition of the n-back task scan. Because we were interested in state changes, we constructed transition matrices that ignore the potentially independent effects of state persistence, or autocorrelation, and only capture the probabilities of moving to new states; that is, the *ij*th element of the transition matrix represents the transition probability between state *i* and state *j* given that a transition out of state *i* is occurring (Fig. 4a, see “Methods” section). As an initial step, we used two null models to confirm previous findings^48^ that brain state transitions are non-random, in that the observed transition probabilities would be unlikely in uniformly random sequences of states and state transitions (Supplementary Fig. 9).

Next, we explored how cognitive load impacts brain state transitions using a non-parametric permutation test to assay for differences between transition matrices computed from resting state scans and from the 2-back condition of the n-back task. We hypothesized that we would see more transitions from states driven by sensory cortex activation into states driven by activation in executive control and attention areas, reflecting reception, integration, and task-relevant processing of stimuli. Indeed, we found that transitions from VIS+ and VIS− states into the FPN+ state were increased during the 2-back condition compared to rest scans (Fig. 4b). Transitions from DMN+, DMN−, and FPN+ states into VIS+ states were also increased in the 2-back condition, likely reflecting the interruption of ongoing transmodal processing by sensory input. Finally, we tested for associations between 2-back transition probabilities and performance during the 2-back condition. In support of our hypothesis, we found that transitions from the VIS− state to the DMN+ state were negatively associated with performance (Fig. 4d, multiple linear regression, standardized *β*_TP_ = −0.14, df = 873, *t* = − 4.42, *p*_corr_ = 2.24 × 10^−4^), while transitions from the VIS− state to the FPN+ state were positively associated with performance (Fig. 4d, multiple linear regression, standardized *β*_TP_ = 0.14, df = 873, *t* = 4.37, *p*_corr_ = 2.84 × 10^−4^). These results are consistent with prior work positing roles for the FPN and DMN as task-positive and task-negative systems^45^, but suggest that interactions with motor, visual, and salience networks found in the VIS− state may also contribute to working memory. Overall, these findings suggest that specific trajectories in brain activation space are favored during increased cognitive load and may represent task-relevant processing.

### Control properties of white matter networks explain brain state transitions

**Fig. 5:**
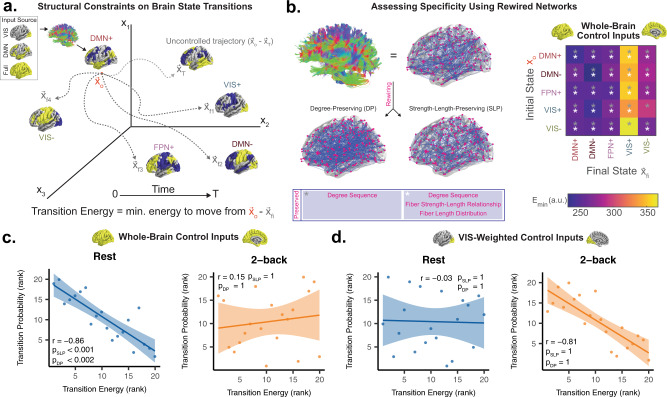
**Linear dynamics along white matter explain brain state transition probabilities**. **a** Schematic demonstrating calculation of minimum control energy needed to move a linear dynamical system defined by white matter connectivity from some initial state **x**_0_ to some final state ${{\bf{x}}}_{{\mathrm{{{f}}}}_{i}}$ over a time horizon *T*. **b** Schematic of network null models (left) preserving different spatial and topologic features of networks defined by white matter connectivity. The energies (E_min_) required to maintain or transition between each state are lower in real brain networks compared to these null models (right). **c** and **d** Spearman correlation between structure-based transition energy prediction (*x*-axis) and empirically derived transition probability (*y*-axis) for resting state (left) and the 2-back condition of the n-back task (right), using inputs weighted evenly throughout the whole brain **c** or weighted positively towards the visual system **d**. Linear fit of rank values are shown due to the monotonic, non-linear relationships in the data.

In the previous section, we described how the presence of cognitive demands and sensory inputs leads the brain towards certain trajectories in state space. However, it is not well understood how the static white matter connectome contributes to these divergent dynamics. Here, we modeled the influence of structure on brain activity as the time-evolving state of a linear dynamical system defined by white matter connectivity. By applying tools from network control theory (Fig. 5a; see “Methods” section, subsection “Network control theory” and Supplementary Information, subsection “Calculating transition energy using control theory”), we calculated the transition energy as the minimum input energy needed to transition between every pair of the empirically observed brain states. In all calculations, we allowed the inputs to come from all brain regions, weighted either uniformly or towards a particular cognitive system^43^. Using this framework, we tested a series of hypotheses unified under the notion that the brain prefers trajectories through state space requiring minimal input energy given structural constraints.

First, we hypothesized that the brain is optimized to support the observed brain states and state transitions with relatively little energy. We measured brain state stability as persistence energy, or the energy needed to maintain each state. In a single representative human structural brain network^49,50^ (see “Methods” section for details), we compared the transition and persistence energies for real structural connectivity (Fig. 5b) and for two null models based on the group average human structural brain network: (1) a null model that preserves only degree sequence in the networks^51^ (Deg. Pres., DP), and (2) a null model that preserves degree sequence, edge length distribution, edge weight distribution, and edge weight–length relationship^41^ (strength–length preserving, SLP). Compared to the DP null model, transition and persistence energy were always lower in the group average SC (Fig. 5b, all *p*_corr_ < 0.001). Compared to the SLP null model, every single persistence energy value and all but two transition energy values were lower in the group average SC (Fig. 5b, *p*_corr_ < 0.001). Finally, we found that the energy required to maintain the DMN+ state was lower than a set of null states with similar spatial covariance^40^ (Supplementary Fig. 10). Collectively, these findings suggest that unique geometric and topological features of white matter networks allow for low-energy transitions and maintenance of the empirically observed functional states.

Next, we hypothesized that the brain prefers trajectories through state space that require little input energy to achieve in a dynamical system defined by white matter connectivity. To test this hypothesis, we computed the Spearman correlation between transition energy values and transition probabilities observed during resting state scans and during the 2-back condition of n-back task scans (Fig. 5c and d). When inputs are evenly weighted throughout the whole brain (Fig. 5c), transition energy values are strongly anticorrelated with resting state transition probabilities and weakly correlated with 2-back transition probabilities. Importantly, the energy estimates from real structural connectivity were more strongly anticorrelated with resting state transition probabilities than energy estimates from null models or transition distance in state space alone (Spearman’s *r* = −0.86, *p*_SLP_ < 0.001, *p*_DP_ < 0.001, Supplementary Fig. 11b). When inputs are biased towards the visual system^43^ (Fig. 5d), transition energy values are strongly anticorrelated with 2-back transition probabilities (Spearman’s *r* = −0.81) and weakly correlated with resting state transition probabilities (Spearman’s *r* = −0.03). However, this result was primarily explained by transition distance in state space, rather than the effects of structure (*p*_SLP_ = 1, *p*_DP_ = 1). Overall, these findings suggest that linear diffusion of brain activity along white matter tracts constrains brain state transitions at rest, and that the distribution of inputs to the brain is an important factor in the brain’s progression through state space.

### Brain state dynamics and control energies are associated with age

Developmental changes in white matter, gray matter, functional networks, and task-related activations accompany changes in behavior and cognition^5,52–55^. However, it is unclear how state space trajectories and their supporting structural features contribute to these cognitive and behavioral changes. Given that the spatiotemporal brain dynamics identified by our approach have clear structural underpinnings, we hypothesized that these dynamics change throughout normative neurodevelopment in support of emerging cognitive abilities^56,57^.

**Fig. 6:**
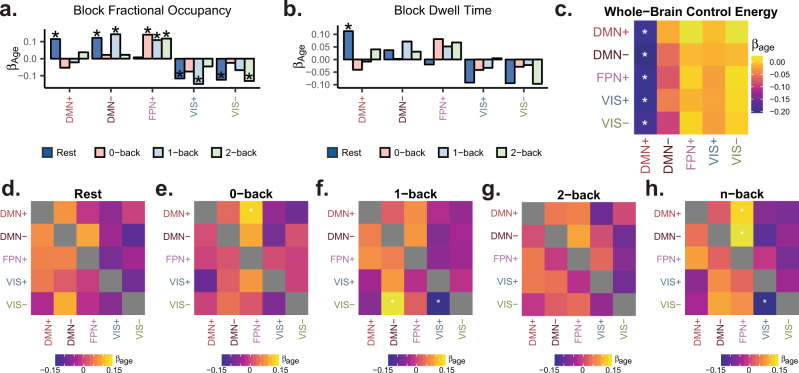
**Brain state dynamics and control energies are associated with age**. **a** and **b** Standardized linear regression *β* weights for age as a predictor of fractional occupancy **a** or dwell time **b** in each state during rest and during each condition of the n-back task. *p_corr_ < 0.05 after Bonferroni correction over 20 state transitions. **c** Standardized linear regression *β* weights for age as a predictor of minimum control energy required to transition between each pair of states. *p_corr_ < 0.05 after Bonferroni correction over 20 state transitions. **d**–**h** Standardized linear regression *β* weights for age as a predictor of transition probabilities during resting state scans **d**, 0-back **e**, 1-back **f**, and 2-back **g** conditions of the n-back task, and the entire n-back task scan **h**. *p_corr_ < 0.05 after Bonferroni correction over 100 state transitions shown in panels **d**–**h**.

We used multiple linear regression to ask whether age was associated with state dwell times and fractional occupancies while controlling for brain volume, handedness, head motion, and sex as potential confounders. Interestingly, we found that fractional occupancies in FPN+ and DMN+ states exhibited context-dependent associations with age (Fig. 6a). FPN+ fractional occupancy increased with age for all blocks of the n-back task (Fig. 6a; multiple linear regression, 2-back standardized *β*_age_ = 0.12, df = 873, *t* = 3.40, *p*_corr_ = 0.014) and not rest, while DMN+ fractional occupancy increased with age for rest only (Fig. 6a; multiple linear regression, standardized *β*_age_ = 0.12, df = 873, *t* = 3.59, *p*_corr_ = 0.015). The relationships between dwell time and age followed similar but weaker trends to those observed with fractional occupancy, with the exception of resting state DMN+ state dwell time which increased with age. We also found that the minimum control energy required to undergo all transitions that terminated in the DMN+ state decreased with age (Fig. 6c; all *p*_corr_ < 0.05). This finding suggests that age-associated structural changes allow individuals to coherently activate the default mode network with greater ease, and is consistent with the observation that DMN+ dwell time and fractional occupancy increase with age at rest.

We also assessed whether transition probabilities were associated with age. Using multiple linear regression, we tested for relationships between transition probabilities or transition energy values and age, while controlling for brain volume, handedness, head motion, and sex. Similar to the context-dependent associations with age that we observed with fractional occupancy, we found that transition probabilities were differentially associated with age across the conditions of the n-back task (Fig. 6e–h). The probability of transitions from both DMN− (Fig. 6f; multiple linear regression, standardized *β*_age_ = 0.15, df = 873, *t* = 4.33, *p*_corr_ = 1.7 × 10^−3^) and DMN+ (Fig. 6f; standardized *β*_age_ = 0.13, df = 873, *t* = 3.68, *p*_corr_ = 0.025) into FPN+ during the n-back task increased with age. This observation is particularly interesting in light of previous work implicating the DMN and FPN in increasing working memory performance across development^54^. Specifically, this result provides evidence for the importance of direct switching between DMN and FPN states, as opposed to deactivation and activation without any temporal constraints. Overall, these findings suggest that task-oriented and spontaneous brain dynamics involving the DMN and FPN may mature through independent processes.

## Discussion

In the present study, we examined the temporal sequence of whole-brain activity patterns in individuals during rest and task, and demonstrate a structural basis for large-scale brain activity patterns and their dynamic temporal evolution. Using a diverse array of techniques from network neuroscience, dynamical systems, and network control theory, we generated new insights into the complex relationship between brain structure, spatiotemporal patterns of brain activity, neurodevelopment, and behavior.

Cognitive functions are often represented as brain activity patterns^19^, but substantially less is known about how sequences of activity patterns may represent links between cognitive functions. In this paper, we considered each fMRI image acquisition to be a point in a high-dimensional state space whose axes correspond to regional activity. Next, we identified brain states as frequently visited locations in this space comprised of combinations of active and inactive brain networks^43^. Finally, we described the directional trajectories between these states in time as state transitions. Our work adds to a body of literature suggesting that coactivation of brain networks at relatively short temporal scales evidences rich functional interactions supporting behavior^6,9,11^. For instance, we found that the brain state transition probabilities observed at rest were strongly modulated by cognitive demand. During resting state scans, in which external stimuli are constant over time, transitions likely occur spontaneously, while during n-back task scans, transitions are likely caused by a combination of spontaneous fluctuations, stimulus-evoked activity in primary sensory areas, and task-related activity changes in higher order association areas. In the n-back task, we found more frequent transitions into states driven by coactivation of sensory systems from states involving coactivation of higher order association areas when compared to the resting state. This finding was present in two independent samples (PNC and HCP) with different task structure and is consistent with increased top-down modulation^20^ of sensory input during task performance.

We also found that certain trajectories in state space were related to task performance. The VIS− state occurred more frequently during the n-back task and is composed of visual cortex suppression alongside mixed dorsal and ventral attention network activation, consistent with top-down suppression of sensory cortex^20^. While its occupancy alone was not related to task performance, transitions from VIS− to FPN+ or DMN+ were positively and negatively associated with performance, respectively. These findings suggest that early stimulus processing followed by manipulation^58^ of task-relevant information facilitates accurate performance, while stimulus processing followed by internally directed cognition^46^ is detrimental to performance. More broadly, this result suggests that the paths through which activation patterns are reached are important, in addition to the activation patterns themselves.

Our major contribution to cognitive neuroscience and applied network science lies in describing how linear diffusion of activity along a static white matter architecture constrains trajectories through brain activation space at rest. We hypothesized that the state space of brain activity could be explained by two components: linear diffusion of activity along white matter tracts^59^ and some nonlinear inputs, which include but are not limited to neuronal membrane dynamics, metabolic factors, and external stimuli. Under this model, we solved for the magnitude of these nonlinear inputs required to maintain and transition between brain states, given the constant constraint of linear diffusion of activity along white matter tracts.

Using this approach, we found that the brain empirically prefers trajectories in state space requiring the least energy needed to overcome structural constraints for a given set of inputs. Specifically, when we modeled uniformly weighted inputs or input weighted towards the DMN, the resulting transition energy values best explained resting state transition probabilities, possibly reflecting a regime with heterogeneous drivers centered around the DMN^46^. As expected, these transition energies did not explain transition probabilities during the 2-back condition, likely due to task-derived inputs which were not explicitly modeled. Indeed, when we weighted system input towards the visual system to account for the frequent delivery of visual stimuli, we were better able to explain 2-back transition probabilities. While this finding represents constraints of distance alone and not white matter topology, it quantitatively explains how that stimulus-derived input alters the state-space trajectories of the brain. Future investigations may resolve the effects of structure on task dynamics using data-driven approaches that attempt to recover the full set of task-related inputs^60,61^.

Unlike previous time point level fMRI analyses^6,9,11^, our method unambiguously labels every time point in every subject for rest and n-back as belonging to a discrete, common state. We intentionally designed our method in this way to make comparisons across contexts and across subjects throughout different developmental stages^62,63^. Indeed, these comparisons revealed context-specific associations between age and brain state dynamics, suggesting that as brain structure develops, multiple trajectories through state space are supported. Our study offered insights into previously unexplored time-resolved brain dynamics in normative neurodevelopment. Neuropsychiatric illnesses such as schizophrenia, autism, epilepsy, and ADHD are increasingly considered developmental disorders, and therefore it is critical to understand the maturation of brain dynamics in healthy youth. Previous studies have shown that structural and functional changes in the DMN and FPN accompany normal cognitive development^64–66^. Here we contribute to our understanding of these networks by demonstrating context-dependent associations between age and DMN and FPN state dynamics (Fig. 6a, d and e). Interestingly, both fractional occupancies and state transition probabilities exhibit context-dependent associations with age, with DMN+ fractional occupancies increasing with age at rest only (Fig. 6a), FPN+ fractional occupancies increasing with age in n-back only (Fig. 6a), and DMN to FPN+ transitions increasing with age during task only (Fig. 6e). However, like other cross-sectional studies of the relationship between brain function and age^54,67,68^, we found relatively small effects of age on individual measures. Consistent with the finding of DMN+ fractional occupancy increasing with age, we also found that the predicted energy of transitioning into the DMN+ state from all other states decreased with age. However, we did not find a significant relationship between DMN+ transition energy values and DMN+ fractional occupancy across subjects. Future work should explore how the relative architecture of control energies within subjects may explain a bias towards certain trajectories over others.

### Methodological limitations

We acknowledge that a limitation of this study was a focus on discrete brain states with common spatial activity patterns across subjects rather than a combination of continuously fluctuating spatial modes of brain activity^4,6^. While BOLD fMRI fluctuations are generally thought to be continuous in nature, several studies have found evidence for temporally “bursty” whole-brain activity patterns^17,69,70^, consistent with discrete, event-like dynamics. Additionally, a discrete model is consistent with the notion of that specific patterns of neural activity and connectivity represent information^71,72^. *k*-means clustering and principal component analysis (PCA) are two related techniques^73^ providing discrete and continuous solutions, respectively. We observed that our cluster centroids separately corresponded to the positive and negative versions of the spatial principal components (PCs) of our data, suggesting that these two approaches would likely reveal convergent findings. However, our discrete approach constituted a major strength of the study, because it allowed us to study sequences of brain activity using approaches from stochastic process theory^74^, including calculating transition probabilities. The discrete model also allowed us to use network control theory to relate structural connectivity to brain activity patterns at each image acquisition, while PCA models each image acquisition as a mixture of lower dimensional components. Assuming that our clusters are comparable to positive and negative loadings on PCs, then the clustering approach employed here essentially provides a data-driven thresholding to identify time points when the brain moves from a loading on one spatial mode to another, while providing information about the direction (positive or negative) of loading on those components involved in those transitions. An arbitrary threshold would be required to identify these time points relative to PC time courses. Discretization also allows us to easily identify, for example, that a negative loading on PC *i* tends to be followed in time by a negative loading on PC *j*, whereas the analagous continuous approach of computing a lagged correlation value between PC time courses would not by itself discern between positive following positive and negative following negative. Importantly, our approach also inherently accounts for the temporal autocorrelation within the BOLD signal^75^ by measuring state transitions while excluding state persistence.

We also demonstrated that *k* = 5 yields stable cluster partitions robust to outliers (Supplementary Fig. 2), and our results were consistent for multiple values of *k* (Supplementary Fig. 12). These findings suggest that *k* = 5 is not a “magic number” of states visited by the brain, but rather one scale at which the brain can be studied. One can always identify *k* clusters from a dataset; however, in our case, we showed that the observed cluster centroids depended on the covariance structure of the BOLD data that was not explained by autocorrelation (Supplementary Fig. 3), suggesting that the studied states reflect non-trivial coactivations between brain regions. Nevertheless, discretizing a system that exhibits continuous behavior will remove information, some of which may be important for understanding brain–behavior relationships.

The relatively low sampling rate (TR = 3 s) likely limited our ability to resolve fast changes in brain activity. Nevertheless, we were able to resolve the effects of specific brain state transitions on behavior (Fig. 4d, g). Additionally, there likely exist meaningful differences in individual brain state topographies^76,77^ that certainly warrant further investigation, but could not be studied convincingly here due to the relatively small number of time frames acquired for each subject. To partially address these limitations, we reproduced key findings in a second parcellation (Supplementary Fig. 5) and an independent sample with a higher sampling rate and no global signal regression (Supplementary Fig. 6).

### Future directions

The novel approaches in this study pave the way for many future studies to continue to elucidate how a static structural connectome can give rise to complex, time-evolving activity patterns important for cognition. An intuitive and important application of our approach lies in the field of neurostimulation, where clinicians aim to implement targeted changes in the temporal evolution of brain activity patterns^27,30,78^ to alleviate symptoms of neuropsychiatric illness. In particular, network control theory and data-driven estimation of brain states are a powerful combination for this purpose. However, before this application can be realized, the robustness of these models at the level of individual subjects must be confirmed. One could similarly ask whether individual differences in structural connectivity explain variance in brain state dynamics, and thus response to neural stimulation. Application of these methods to electrophysiologic data could help to validate our findings and elucidate more complex neural dynamics that are not reflected in the slow fluctuations of hemoglobin oxygenation captured by BOLD fMRI^79,80^.

Targeted, model-informed brain stimulation^1,27,78^ will likely need to account for interactions between exogenous input and endogenous dynamics^60,61^. Recent evidence^4^ implicates ascending neuromodulatory inputs in the brain’s progression through state space. Release of neuromodulators can be driven by external stimuli or spontaneous neural activity^81^, and therefore may serve as both an important mediator of external inputs and a critical aspect of endogenous dynamics. Ultimately, a model that integrates the dynamic and static interactions between brain structure, neuromodulators, fast ionotropic neurotransmission, and exogenous inputs might allow clinicians to solve for inputs that effect beneficial changes in brain activity and connectivity. Nonlinear neural mass models of brain activity hold substantial promise for this purpose^78,82^ and in the future could be used to more deeply probe the structural constraints on brain dynamics identified here.

## Methods

### Participants

Resting state fMRI, n-back task fMRI, and diffusion tensor imaging (DTI) data were obtained from *n* = 1601 youth who participated in a large community-based study of brain development, known as the PNC^31^. The institutional review boards of the University of Pennsylvania and the Children’s Hospital of Philadelphia approved all study procedures. Participants had been previously enrolled in a study at the Center for Applied Genomics, and participants and/or their parents provided informed consent (assent) to be re-contacted for participation in additional studies, including the PNC. Here we study a sample of *n* = 879 participants between the ages of 8 and 22 years (mean = 15.9, s.d. = 3.3, 386 males, 493 females) with high quality diffusion imaging, rest BOLD fMRI, and n-back task BOLD fMRI data. Our sample only contained subjects with low estimated head motion and without any radiological abnormalities or medical problems that might impact brain function (see Supplementary Information for detailed exclusion criteria). Details about imaging parameters, task design, and image preprocessing can be found in the Supplementary Information.

### Unsupervised clustering of BOLD volumes

BOLD fMRI activity patterns are known to represent information content^18^, information processing^19,20^, and attention to stimuli^20^. Here, we use a discrete model as a simplification of brain dynamics^48^, in which we view repeatedly visited locations in regional activation space to be neural representations of cognitive states, or “brain states” for simplicity. In order to ultimately characterize the progression of these brain states from one time point to the next, and by extension the progression of the brain through regional activation space, we began by concatenating all functional volumes into one large data matrix^4^. Specifically, we took all brain-wide patterns of BOLD activity from the resting-state scan and from the n-back task scan from all subjects, and we placed them into a matrix **X** with *N* observations (rows) and *P* features (columns). Here, *P* is the number of brain regions in the parcellation (462), and *N* is the number of subjects (879) × (120 resting state volumes + 225 n-back task volumes), summing up to *N* = 303,255.

To determine the brain states present in these data, we performed 20 repetitions of *k*-means clustering for *k* = 2 to *k* = 11 using Pearson correlation as the algorithm’s measure of distance^7,8,35^. Because we aimed to study the temporal progression between coactivation patterns using a *k* × *k* transition probability matrix, and our resting state scans contained 120 frames, *k*^2^ must be <120 in order to theoretically observe each transition at least once. Therefore, we chose *k* = 11 (*k*^2^ = 121) as our maximum possible value of *k*. After selecting *k* = 5, we chose to consider the partition with the lowest error out of all 20 repetitions for subsequent analyses. To identify the optimal number of clusters *k*, we assessed the variance explained by the lowest error solution of the clustering algorithm at each value of *k* from 2 to 11, and the gain in variance explained for a unit increase in *k*. The variance explained by the clustering algorithm is defined by the ratio of between-cluster variance to total variance in the data (within-cluster variance plus between-cluster variance)^35,83^. We also intended to make cross-subject comparisons of state dynamics as continuous measures, so it was important to use partitions that identified brain states that were common across all subjects, rather than identifying many different states that were each only represented in a few subjects.

We observed that the variance explained by the clustering algorithm began to taper off after *k* = 5 (Supplementary Fig. 2a), and the additional variance explained for each unit increase in *k* after 5 was  <1% (Supplementary Fig. 2b). Additionally, *k* values >5 produced states that were not all represented in every subject (Supplementary Fig. 2c). To avoid using an unnecessarily large number of states while maintaining inter-subject correspondence in state presence, we chose *k* = 5. To further validate the choice of *k* = 5, we evaluated the split reliability of the partition at this resolution (Supplementary Fig. 2d–f). This analysis showed that cluster centroids and transition probability matrices were highly similar between independently clustered subject samples (see Supplementary Methods for details). Another recent paper^35^ found a similar drop off in additional variance explained at *k* = 6 instead of *k* = 5. Key findings are reproduced at *k* = 6 in the supplement and at *k* = 5 for a second parcellation.

### Analysis of spatiotemporal brain dynamics

After using *k*-means clustering to define discrete brain states, we generated names for each state using the maximum cosine similarity to binary vectors reflecting activation of communities in an a priori defined 7-network partition^43^; names were generated separately for maximum cosine similarity of positive and negative state entries. These names only serve as a convenient way of referring to clusters instead of their index (i.e., 1−*k*), and have no impact on any analyses. Next, we computed subject-level state fractional occupancy as the percentage of volumes in each scan that were classified as a particular state. Additionally, we computed subject-level state dwell time as the mean length of consecutive runs of each state. We defined the transition probability between state *i* and state *j* to be the probability that *j* is the next new state occupied after state *i*. This can also be equivalently framed as the probability of a specific state transition occurring given that some state transition is occurring. We chose this metric in order to understand state transitions without bias from potentially independent effects of state dwell time or autocorrelation. Operationally, this computation was performed by reducing the empirically obtained state sequences to a new sequence (e.g. [1 1 1 2 2 3 2 2] becomes [1 2 3 2]) in which the dwell time of every state is equal, and then computing the probability of state *j* following state *i*. In the supplement, we also compute the transition probability between two states as the probability of transitioning from state *i* at time *t* to state *j* at time point *t* + *t*_r_ given that the current state is *i*, where *t*_r_ is the TR of the BOLD scanning sequence (3 s for PNC and 0.72 s for HCP) for the purposes of demonstrating the non-random nature of brain state dynamics.

Finally, in order to assess the context-dependent nature of brain state dynamics, we performed a non-parametric permutation test to compare group-average transition probabilities between the n-back task and the resting state. First, we randomly selected two halves of the full sample. Next, we generated two group-average transition matrices by averaging together resting state transition matrices from one half and n-back transition matrices from the other half, and vice versa. This procedure was repeated 100,000 times, and we retained the difference between the two halves at every element of the transition matrix. We generated a *p*-value for each element of the transition matrix by dividing the number of times the observed difference between n-back and rest at that element exceeded the null distribution of differences.

### Network control theory

To better understand the structural basis for the observed brain states themselves, as well as their persistence dynamics, we employed tools from network control theory^39,84^. We represent the fractional anisotropy-weighted structural network estimated from diffusion tractography as an *N* × *N* matrix **A**, where *N* is the number of brain regions in the parcellation and the elements **A**_*ij*_ contain the estimated strength of structural connectivity between regions *i* and *j*, where *i* and *j* can range from 1 to *N*. Because diffusion tractography cannot estimate within-region structural connectivity, *A*_*ij*_ = 0 whenever *i* = *j*.

We allow each node to carry a real value, contained in the map ${\bf{x}}:{{\mathbb{R}}}_{\ge 0}\to {{\mathbb{R}}}^{N}$, to describe the activity at each region in continuous time. Next, we employ a linear, time-invariant model of network dynamics:

1 $$\dot{{\bf{x}}}(t)={\bf{Ax}}(t)+{\bf{B}}{\bf{u}}(t),$$

where **x** describes the activity (i.e. BOLD signal) in each brain region over time, and the value of the *i*th element of **x** describes the activity level of region *i*.

After stipulating this dynamical model, we computed the *k* × *k* transition energy matrix **T**_e_ as the minimum energy required to transition between all possible pairs of the *k* clustered brain states, given the white matter connections represented in **A**. See Supplementary Methods for details on computation of minimum control energy and selection of a control horizon. For the purposes of control theoretic simulations, we were interested in exploring the fundamental role of white matter architecture in supporting brain state transitions. Thus, we constructed a single group-representative **A** generated through distance-dependent consistency thresholding^49^ of all subjects’ structural connectivity matrices, a process which has been described in detail elsewhere^50^.

### Developmental and cognitive trends of brain dynamics

After identifying context-dependent brain dynamics at the level of individual frames, we hypothesized that features of these dynamics would change throughout normative neurodevelopment, and moreover that they would map to cognitive performance. To assess potential developmental trends of spatiotemporal brain dynamics, we fit the following model using linear regression:

2 $$D={\beta }_{0}+{\beta }_{a}a+{\beta }_{v}v+{\beta }_{h}h+{\beta }_{{m}_{\mathrm{{{d}}}}}{m}_{\mathrm{{{d}}}}+{\beta }_{s}s+\epsilon ,$$

where *a* is age, *v* is total intracranial volume, *m*_d_ is the mean framewise displacement during rest or n-back scans, *h* is handedness, *s* is sex, *ϵ* is an error term, and *D* is a measure of brain dynamics, such as fractional occupancy, transition probability, or asymmetry. To assess potential relations between cognitive performance and spatiotemporal brain dynamics, we fit the following model using linear regression:

3 $$C={\beta }_{0}+{\beta }_{D}D+{\beta }_{a}a+{\beta }_{v}v+{\beta }_{h}h+{\beta }_{{m}_{\mathrm{{{d}}}}}{m}_{\mathrm{{{d}}}}+{\beta }_{s}s+\epsilon ,$$

where *C* is the overall or n-back block-specific ${d}^{\prime}$ score, which we use as our measure of working memory performance, and all other variables are the same as described above. For all analyses, we applied a Bonferroni correction for multiple comparisons, accounting for tests performed over all states or state transitions within each scan. We chose the Bonferroni-level correction, because it is a conservative approach given that each state’s fractional occupancies and transitions are not fully independent of one another.

### Statistics and reproducibility

In general, we computed statistics comparing metrics of brain dynamics between resting state and n-back task conditions, based on clustering fMRI data obtained during these conditions. These comparisons were Bonferroni-corrected over the number of metrics tested. In order to test the extent to which the clustering solution was reproducible, we repeated the clustering procedure in 500 split-half subsamples of our data and found strong agreement between the two independent split halves. Additionally, custom code for all data analysis is available publicly (see section “Code availability”) to facilitate reproducibility. This code uses MATLAB, R, and python.

### Diversity statement

Recent work in neuroscience^85^ and other fields^86–89^ has identified a bias in citation practices such that papers from women and other minorities are under-cited relative to the number of such papers in the field. Here we sought to proactively consider choosing references that reflect the diversity of the field in thought, form of contribution, gender, and other factors. We used automatic classification of gender based on the first names of the first and last authors^85,90^, with possible combinations including male/male, male/female, female/male, and female/female. Excluding self-citations to the first and last authors of our current paper, the references contain 56.94% male/male, 11.11% male/female, 19.44% female/male, and 8.33% female/female. Relative to the expected proportions in the field of neuroscience, we over-cited or under-cited these categories by the following ratios: −2.49% male/male, 18.20% male/female, −23.75% female/male, and 24.38% female/female. We look forward to future work that could help us to better understand how to support equitable practices in science.

### Reporting summary

Further information on research design is available in the Nature Research Reporting Summary linked to this article.

## Supplementary information

Description of Additional Supplementary Files

Supplementary Data

Supplementary Information

Reporting Summary

## Data Availability

All structural and functional neuroimaging data are available at https://www.ncbi.nlm.nih.gov/projects/gap/cgi-bin/study.cgi?study_id=phs000607.v3.p2. Source data for main text figures can be found at 10.6084/m9.figshare.11911101.v1^91^.

## Code availability

All analysis code is available at https://github.com/ejcorn/brain_states.
