# Supplementary material for: Temporal sequences of brain activity at rest are constrained by white matter structure and modulated by cognitive demands
Source: Commun Biol. 2020 May 22;3:261. doi: 10.1038/s42003-020-0961-x (PMC7244753; doi:10.1038/s42003-020-0961-x)
Supplement: Supplementary file 1 — Description of Additional Supplementary Files [file 42003_2020_961_MOESM1_ESM.pdf]

## Description of Additional Supplementary Files

### Supplementary Data Zip File

**File Name**: Fig2a__OveralClusterCentroids_k5ScanCLaus250Z0final.mat
**Description**: Figure 2a Source Data

**File Name**: Fig2b__YeoSystemAlignment_k5.mat
**Description**: Figure 2b Source Data

**File Name**: Fig3a__FractionalOccupancy.RData
**Description**: Figure 3a Source Data

**File Name**: Fig3b__DwellTime.RData
**Description**: Figure 3b Source Data

**File Name**: Fig3c__RunRate.RData
**Description**: Figure 3c Source Data

**File Name**: Fig3d__nbackblocks.RData
**Description**: Figure 3d Source Data

**File Name**: Fig3e__nBackBlockFODprime.RData
**Description**: Figure 3e Source Data

**File Name**: Fig4a-c__SourceData.mat
**Description**: Figure 4a-c Source Data

**File Name**: Fig4d__TPvs2backDPrime.mat
**Description**: Figure 4d Source Data

**File Name**: Fig5b__ControlEnergyMagnitudeVsNulls.mat
**Description**: Figure 5b Source Data

**File Name**: Fig5c__WholeBrainControl2Back.RData
**Description**: Figure 5c Source Data

**File Name**: Fig5c__WholeBrainControlRest.RData
**Description**: Figure 5c Source Data

**File Name**: Fig5d__VISControl2Back.RData
**Description**: Figure 5d Source Data
**File Name**: Fig5d__VISControlRest.RData

**Description**: Figure 5d Source Data

**File Name**: Fig6c__TransitionEnergyAge.RData
**Description**: Figure 6c Source Data

**File Name**: Fig6d__RestTPAge.RData
**Description**: Figure 6d Source Data

**File Name**: Fig6e__0backTPAge.RData
**Description**: Figure 6e Source Data

**File Name**: Fig6f__1backTPAge.RData
**Description**: Figure 6f Source Data

**File Name**: Fig6g__2backTPAge.RData
**Description**: Figure 6g Source Data

**File Name**: Fig6h__nbackTPAge.RData
**Description**: Figure 6h Source Data
