# Supplementary material for: Temporal sequences of brain activity at rest are constrained by white matter structure and modulated by cognitive demands
Source: Commun Biol. 2020 May 22;3:261. doi: 10.1038/s42003-020-0961-x (PMC7244753; doi:10.1038/s42003-020-0961-x)
Supplement: Supplementary file 3 — Supplementary Information [file 42003_2020_961_MOESM3_ESM.pdf]

Temporal sequences of brain activity at rest are constrained by white matter structure and modulated by cognitive demands

SUPPLEMENTARY INFORMATION

Supplementary Figures

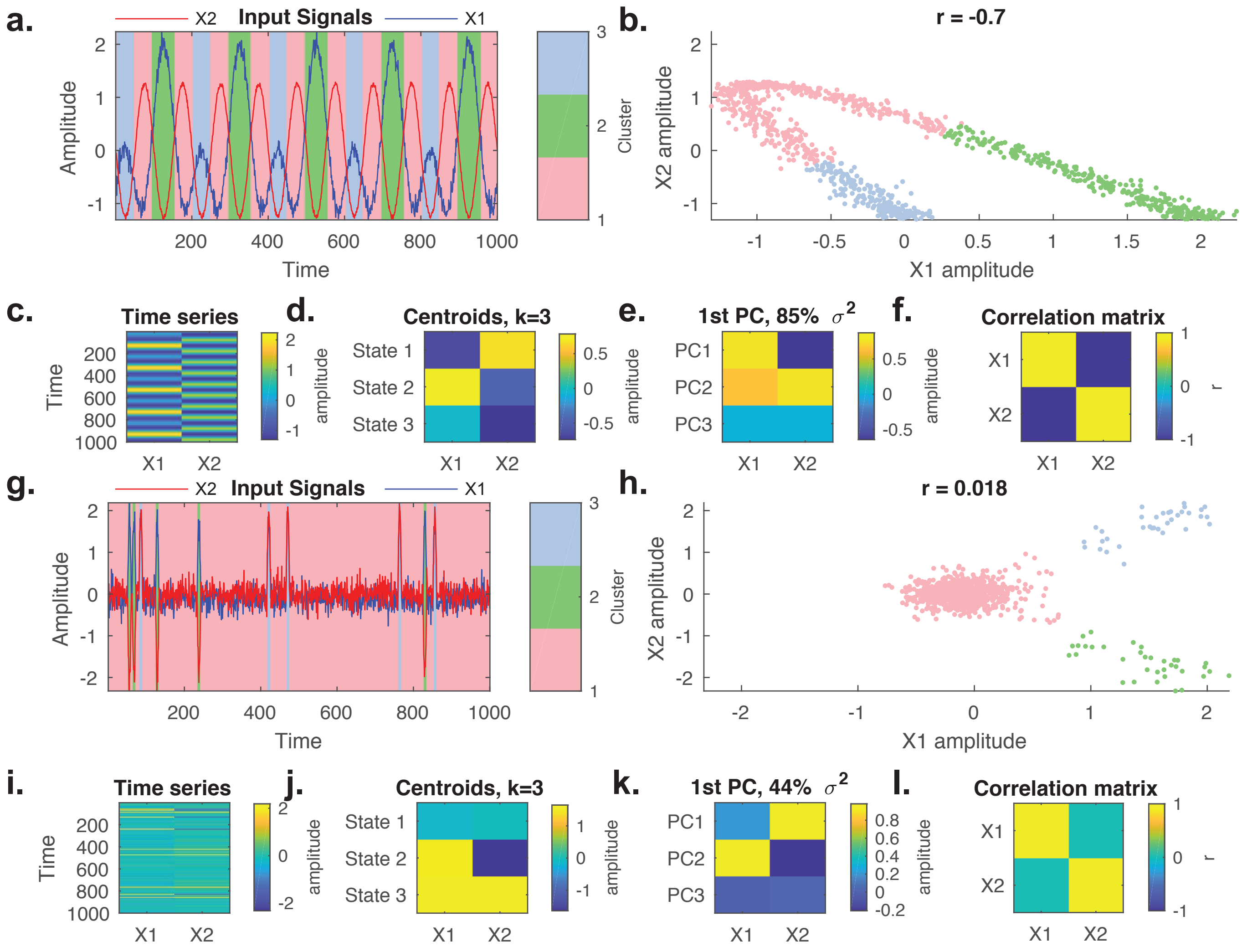

SUPPLEMENTARY FIGURE 1. **Instantaneous coactivation can deviate from temporal correlation patterns.** *(a)* Sinusoidal signal X1 with additive Gaussian noise is plotted in blue. Signal X2, generated by subtracting X1 from a slower sine wave, plus Gaussian noise, is plotted in red. k-means clustering extracts three visually obvious coactivation states (partition shown in light red, green, and blue). *(b)* Plot of X1 amplitude (x-axis) vs. X2 amplitude (y-axis), yielding a Pearson's $r = -0.70$. *(c)* X1 and X2 concatenated into a data matrix, $\mathbf{X_a}$. We concatenated $\mathbf{X_a}$ with two channels of Gaussian noise to generate a 4-column data matrix $\mathbf{X_n}$ in order to use the correlation distance metric. *(d)* Centroids, or "states," obtained through k-means clustering of $\mathbf{X_n}$. Three states emerge; the first state consists of low- and high-amplitude activity in X1 and X2, respectively. The second state consists of low amplitude activity in X2 with near 0 activity in X1. The third state consists of high amplitude activity in X1 with low amplitude activity in X2. *(e)* The first 3 principal component (PC) loadings of $\mathbf{X_a}$. PC1 captures States 1 and 2, but no single PC captures State 3. *(f)* The cross-correlation structure of $\mathbf{X_a}$, which does not reveal the states in panel *(d)*. *(g)* Signal X1, generated by Gaussian noise plus random positive sinusoidal activations is plotted in blue. Signal X2, generated by Gaussian noise plus synchronized coactivation or coinactivation with X1, is plotted in red. k-means clustering extracts three visually obvious coactivation states (partition shown in light red, green, and blue). *(h)* Plot of X1 amplitude (x-axis) vs. X2 amplitude (y-axis), yielding a Pearson's r =0.018. *(i)* X1 and X2 concatenated into a data matrix $\mathbf{X_b}$. We concatenated $\mathbf{X_b}$ with two channels of Gaussian noise to generate a 4-column data matrix $\mathbf{X_m}$ in order to use the correlation distance metric. *(j)* Centroids, or "states," obtained through k-means clustering of $\mathbf{X_m}$. Three states emerge; the first consists of high-amplitude activity in X1 with low-amplitude activity in X2. The second consists of near 0 activity in X1 and X2. The third consists of high-amplitude activity in both X1 and X2. *(k)* The first 3 principal component (PC) loadings of $\mathbf{X_b}$. PC1 does not exist within the data; PC2 mirrors State 1; and both States 2 and 3 are not reflected in any PCs. *(l)* Cross-correlation structure of $\mathbf{X_b}$, which does not trivially reveal the cluster centroids in panel *(d)*.

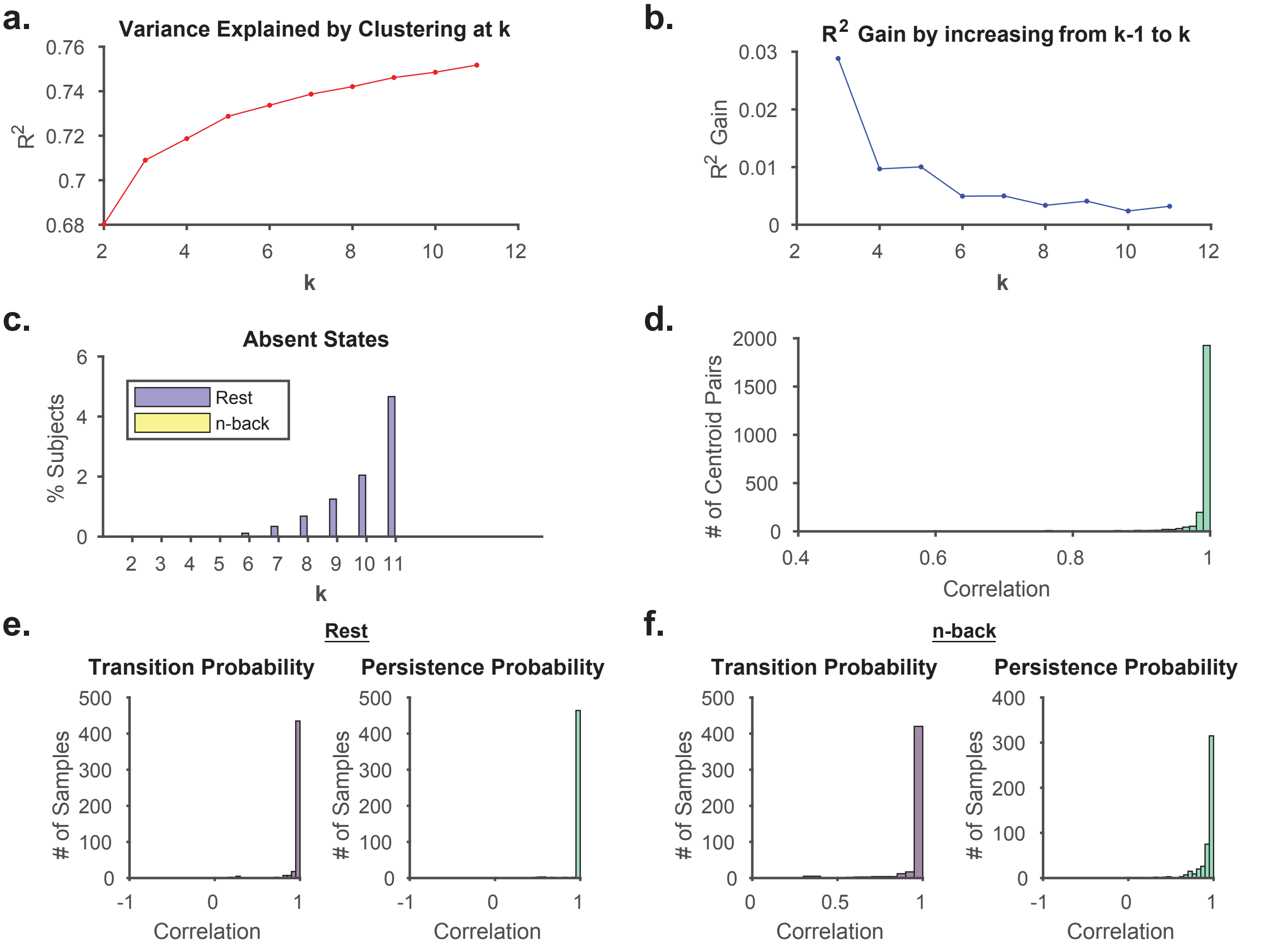

SUPPLEMENTARY FIGURE 2. **Choosing the number of clusters.** *(a)* Variance explained by clustering BOLD data for $k = 2$ to $k = 11$. *(b)* Gain in variance explained by k-means clustering when k is increased from $k - 1$ to k. Less than 1% of additional variance explained is added for $k > 5$. *(c)* Percentage of subjects missing at least one state for rest and n-back. At $k > 5$, states begin to be incompletely represented across subjects. *(d-f)* Split-halves validation of cluster centroids *(c)*, and state transition and persistence probabilities for rest and n-back *(d-e)*. Pearson correlation coefficients between transition and persistence probabilities for each pair of split cluster partitions are predominantly $r > 0.99$, thus showing a high degree of robustness to sample composition in estimating brain states and their dynamics.

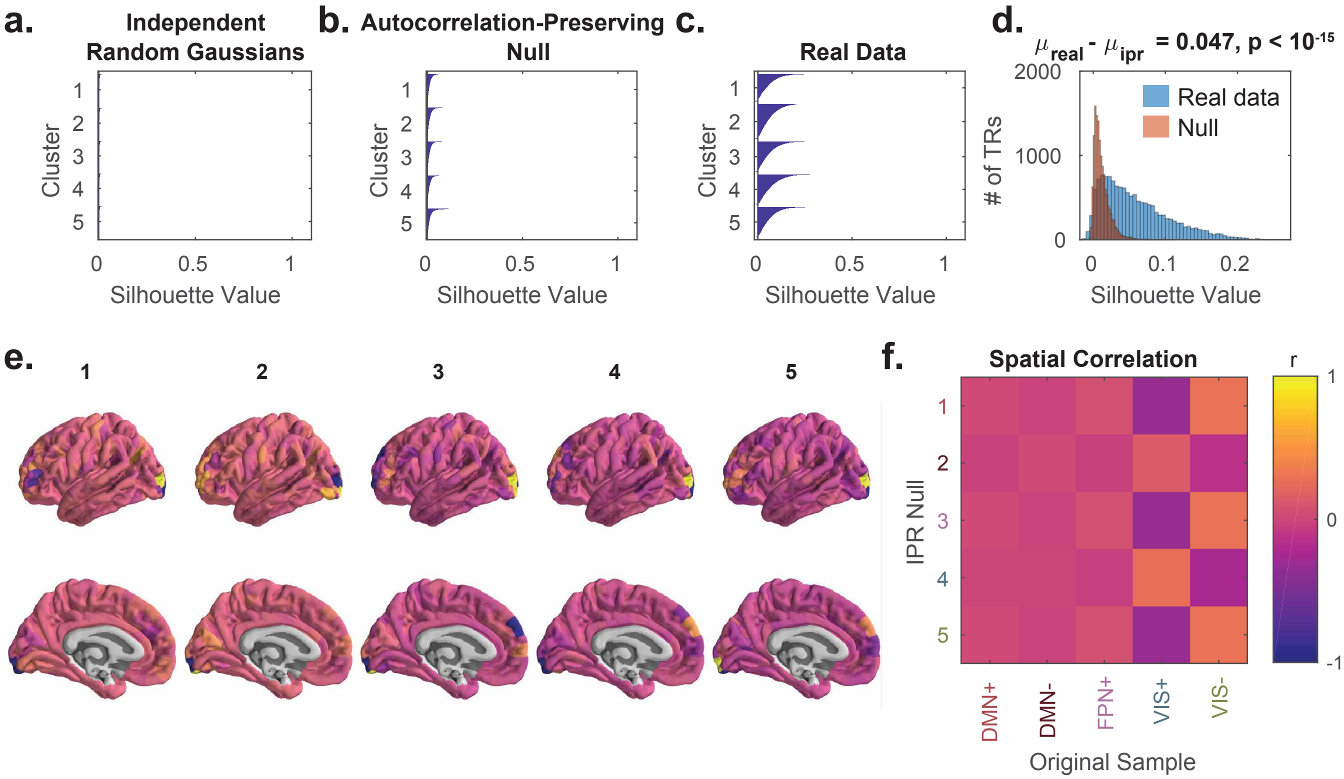

SUPPLEMENTARY FIGURE 3. **BOLD data exhibits clustering in regional activation space.** We performed k-means clustering on rest and n-back data from the $n = 879$ sample studied in the main text at k=5 using the correlation distance in a 462-region activation space. Here we show silhouette scores for rest and n-back time points from 40 random subjects; however, results are consistent across random sets of 10 subjects, suggesting marked reliability. Silhouette scores range from -1 to 1 and are computed for each data point, with 1 indicating that a data point is closer to members of its assigned cluster than to members of the next closest cluster, 0 indicating equidistance between the assigned cluster and the closest cluster, and -1 indicating that the data point is closer to another cluster. *(a)* Silhouette values for data points from 462 independent, normally distributed channels. *(b)* Silhouette values for data from an independent phase randomized (IPR) null model applied separately to resting state and n-back BOLD data. This null model preserves regional autocorrelation while eliminating non-stationarities and reducing covariance. *(c)* Silhouette values for actual resting state and n-back BOLD data. *(d)* Distribution of silhouette values across all clusters for a null model preserving autocorrelation (red, μ_{ipr}) and for actual data (blue, μ_{real}). A two-sample t-test confirms that silhouette values are larger in the real data, indicating that the data are clustered in regional activation space beyond what is expected from a signal with the same regional autocorrelations. *(e)* Cluster centroids at $k = 5$ using IPR resting state and n-back BOLD data. *(f)* Spatial correlation between IPR null centroids in panel e (y-axis) and full sample centroids (x-axis).

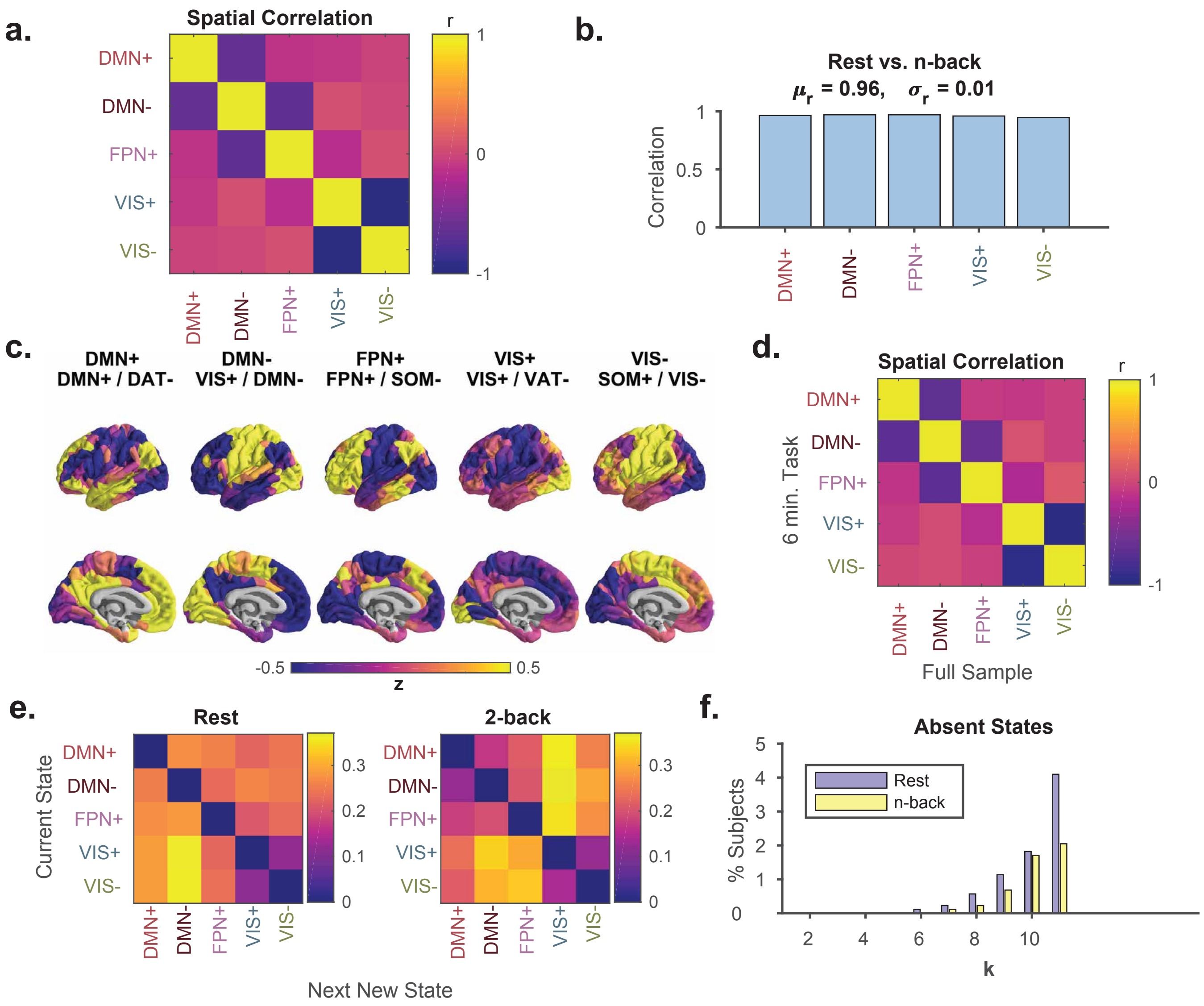

SUPPLEMENTARY FIGURE 4. **Similarity between states in rest and n-back.** *(a)* Spatial correlation between cluster centroids reveals anticorrelation between DMN- and DMN+, between DMN- and FPN+, and between VIS+ and VIS-. *(b)* Spatial correlation between centroids calculated separately for rest and n-back reveal high correspondence, consistent with the identification of recurrent activity patterns common to both scans. *(c)* Cluster centroids computed by including equal amounts of rest and n-back task data as input to the clustering algorithm. Cluster names based on maximum cosine similarity were identical to the full sample centroids. *(d)* Spatial correlation between the 6 minute rest and the 6 minute n-back task cluster centroids and the full sample cluster centroids. Correlation coefficients of $r > 0.99$ were found only on the diagonal, suggesting 1-to-1 correspondence between the two centroid sets. The observed off-diagonal anticorrelations are consist with those observed in the full sample, as shown in panel *(a)*. *(e)* Group average state transition probabilities for rest (*right*) and n-back (*left*) using the 6 minute n-back task cluster partition reveals similar structure and high correlation with full sample state transition probabilities. *(f)* The y-axis shows the percentage of subjects missing at least one state in their time series for rest (*purple*) and for n-back (*yellow*), for values of k on the x-axis ranging from 2 to 18. There existed at least one subject with missing states for all $k > 5$, supporting the choice of $k = 5$ for the main text.

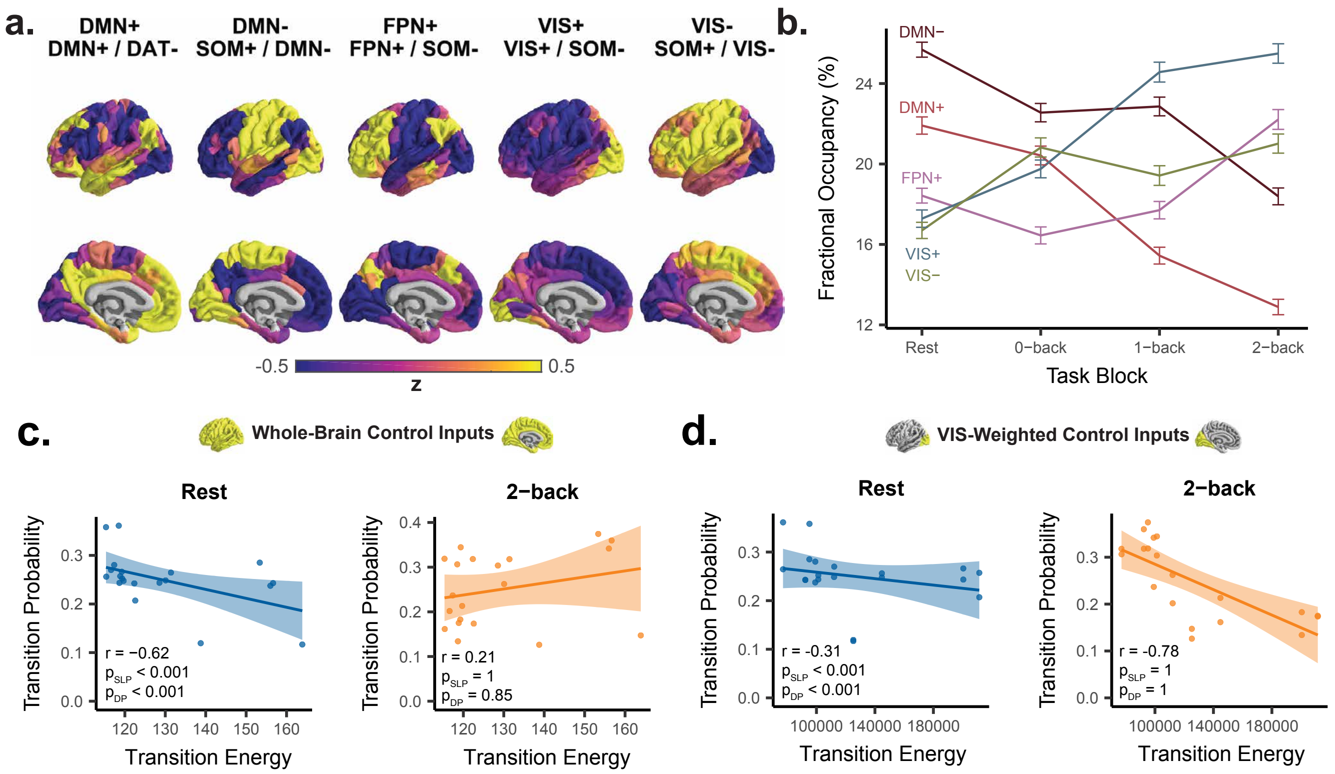

SUPPLEMENTARY FIGURE 5. **Key findings reproduced at $k = 5$ using the 234-node Lausanne parcellation.** *(a)* Cluster centroids at $k = 5$ are similar to that of the 463-node Lausanne parcellation. *(b)* State fractional occupancies change with increasing cognitive load similarly compared to the 463-node parcellation analysis. *(c-d)* Correlation between structure-based transition energy prediction (x-axis) and empirically derived transition probability (y-axis) for resting state (left) and the 2-back condition of the n-back task (right), using inputs weighted evenly throughout the whole brain *(c)* or weighted positively towards the visual system *(d)*.

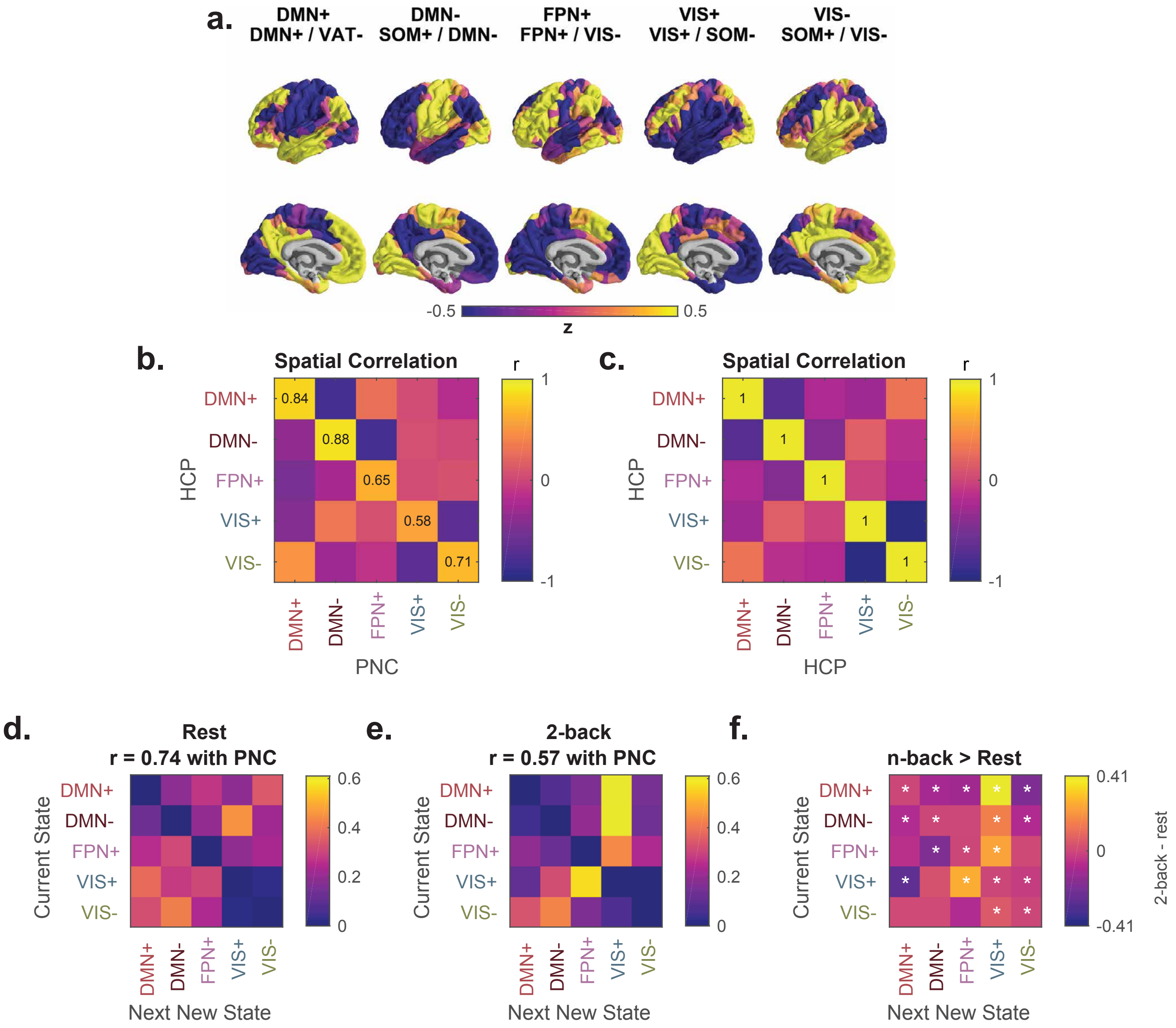

SUPPLEMENTARY FIGURE 6. **Brain states and dynamics in an independent sample with higher sampling rate and no global signal regression.** *(a)* Cluster centroids for clustering of rest and n-back task BOLD data from the Human Connectome Project (HCP) with volumes acquired 4 times as frequently as the PNC and no global signal regression. *(b)* Spatial correlation between centroids for HCP and PNC data sets, is high along the diagonal, allowing for unambiguous matching of brain states between the two samples. *(c)* Spatial correlation between HCP centroids. DMN+ and DMN-, along with VIS+ and VIS-, exhibit strong anticorrelation. *(d-e)* HCP group average state transition probability matrices for rest *(d)* and n-back *(e)* scans. Off-diagonal elements of HCP rest and n-back transition matrices exhibit Pearson correlations of $r = 0.83$ and $r = 0.76$ with the PNC, respectively. HCP persistence probabilities are correlated with PNC persistence probabilities at $r = 0.85$ and $r = 0.85$ for rest and n-back, respectively. *(f)* Non-parametric permutation testing demonstrating differences between the rest and n-back group average transition probabilities and persistence probabilities. Extremes of the color axis indicate statistical significance, with larger values indicating higher transition probabilities in n-back relative to rest. *, Bonferroni-adjusted $p < 0.05$ or $p > 0.95$.

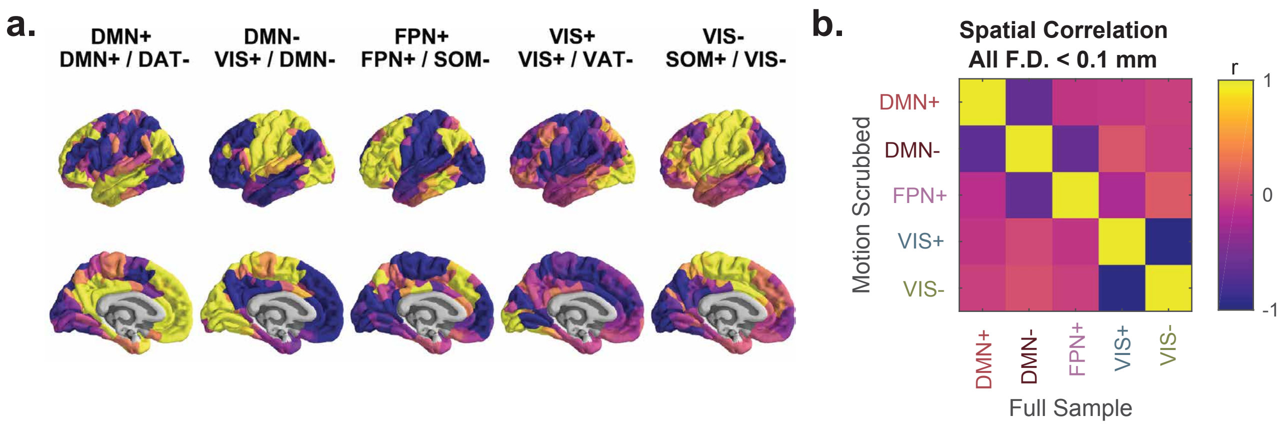

SUPPLEMENTARY FIGURE 7. **Brain states after removing high motion frames.** *(a)* Cluster centroids at $k = 5$ after removing 76,339 volumes with > 0.1mm framewise displacement leaving a total of $N = 226916$ volumes. *(b)* Spatial correlation between motion-scrubbed centroids (y-axis) and full sample centroids (x-axis).

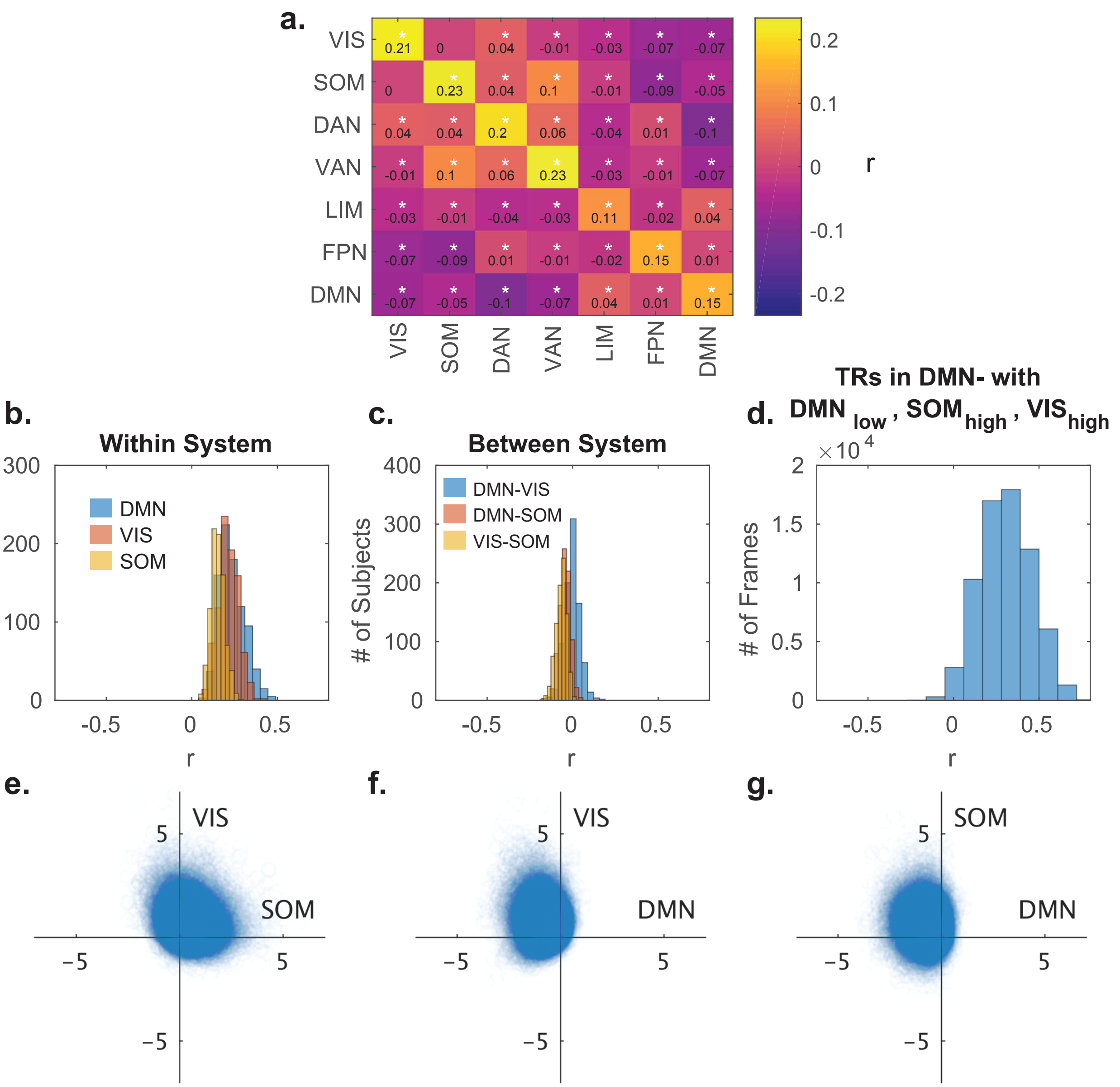

SUPPLEMENTARY FIGURE 8. **Functional connectivity does not fully explain spontaneous coactivation.** *(a)* Group average, mean functional connectivity within and between cognitive systems defined *a priori*[1]. We first computed average functional connectivity matrices by averaging functional connectivity matrices over rest and n-back scans for each subject. Next, we computed the average edge strength within and between regions in each *a priori* defined system to yield mean within- and between-system functional connectivity. Finally, we computed the group average mean within- and between-system functional connectivity across the $n = 879$ subjects from the main text, the values of which are displayed in black text overlaying each heatmap element. *, $p < 0.05$ for a one-sample t-test comparing the mean of the distribution of correlations across subjects to 0. *(b)* Mean temporal correlation values within regions belonging to the default mode network (DMN), visual system (VIS), and somatomotor system (SOM) show positive correlations within systems on average. *(c)* Mean temporal correlations between DMN, VIS, and SOM regions show weakly negative (DMN-VIS, DMN-SOM) or near 0 (VIS-SOM) correlations on average. *(d)* Histogram of TRs (individual BOLD frames) exhibiting activity patterns with low activity in the DMN and high activity in the SOM and VIS systems, as measured by correlation with a binary indicator vector. Despite low temporal correlations, we still find activity patterns with spatial anticorrelation between these systems. *(e-g)* BOLD data in regional activation space. Scatter plots where points are individual BOLD TRs in the DMN- cluster and axes reflect the mean activity across regions in the DMN, VIS, or SOM for each TR, shown in 2 dimensions for ease of visualization. These plots show that TRs in the DMN- cluster (Fig. 2a) have simultaneous high activity in VIS and SOM (panel *d*) in addition to low activity in the DMN (panel *e-f*).

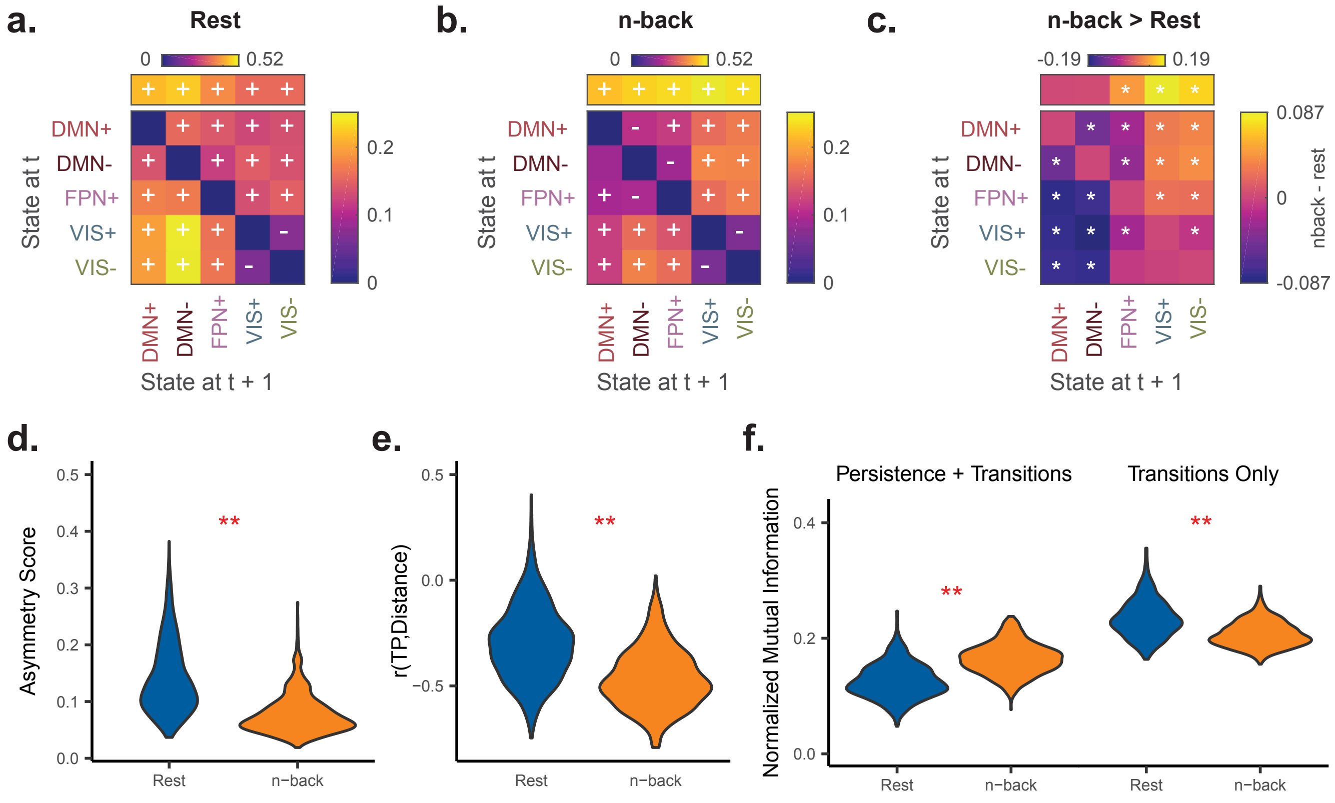

SUPPLEMENTARY FIGURE 9. **Brain state transitions are context-dependent and non-random.** *(a-b)* Group average state transition probability matrices for rest and n-back scans. Overlayed + or - indicates $p_{\mathrm{corr}} < 0.05$ for transitions occurring more or less, respectively, than expected under an appropriate random null model, after Bonferroni correction over 50 tests (20 transition probabilities and 5 persistence probabilities for rest and n-back). Persistence probabilities are removed from the diagonal and depicted above the transition matrix. *(c)* Non-parametric permutation testing demonstrating differences between the rest and n-back group average transition probability matrices. *, $p_{\mathrm{corr}} < 0.05$, after Bonferroni correction over 25 tests: 20 transition probabilities and 5 persistence probabilities. *(d)* Subject-level distributions of matrix asymmetry scores demonstrate that resting state transition probabilities are asymmetric relative to n-back. *(e)* Subject-level distributions of the correlation between transition probabilities and Euclidean distance between states for rest (left) and n-back (right). *(f)* Single-frame lagged, normalized auto mutual information for rest and n-back computed with full state time series *(left)* or transition sequence only *(right)*. **, $p < 10^{-15}$. Paired t-tests were used in panels *(d-f)*. *TP*, transition probability.

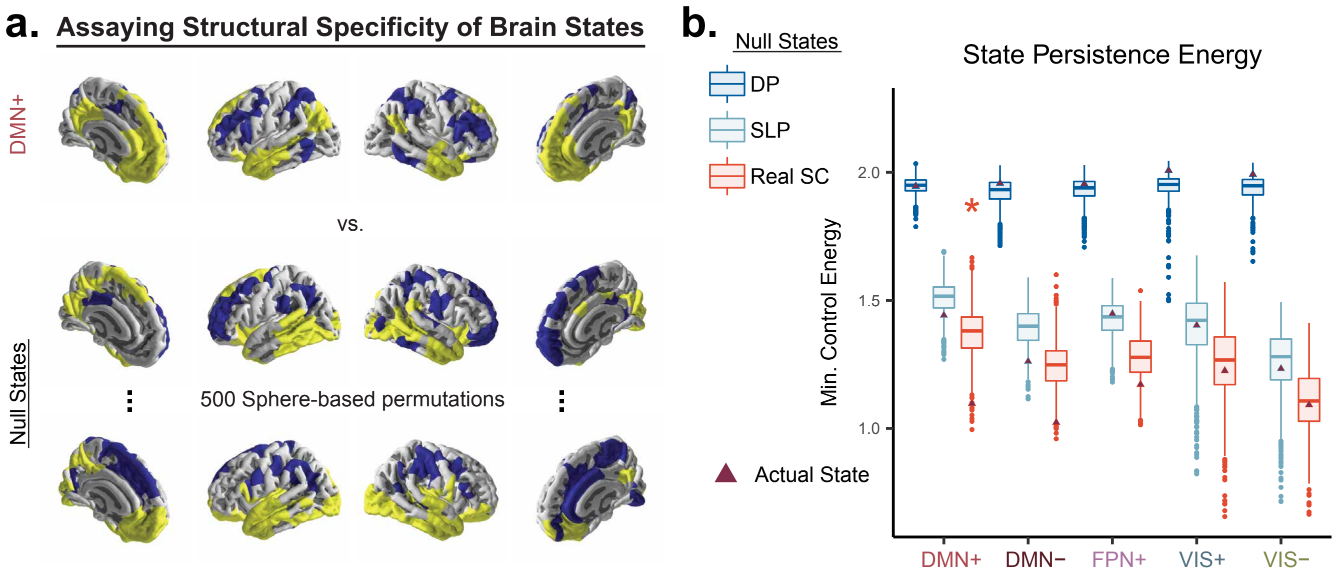

SUPPLEMENTARY FIGURE 10. **Spatial and topological properties of brain structure facilitate selective brain state stability.** *(a)* Construction of null states preserving symmetry and spatial clustering of activity using sphere-based permutation[2]. We compare the minimum control energy required to maintain the brain in each state (persistence energy) relative to spatially permuted states. *(b)* We computed persistence energy for each state and its permuted variants in group average SC (orange) and found that the DMN+ state required less energy to persist than its permuted variants. We performed the same test in two null models: a null model preserving topology (blue) and a null model preserving both topology and spatial constraints (light blue). Orange *, $p_{\mathrm{corr}} < 0.05$ after Bonferroni correction over each of the 5 states, separately for each null model. *(Deg. Pres.)*, degree distribution-preserving null model[3]. *Space Pres.*, degree sequence, edge weight and length distribution and relationship preserving null model[4].

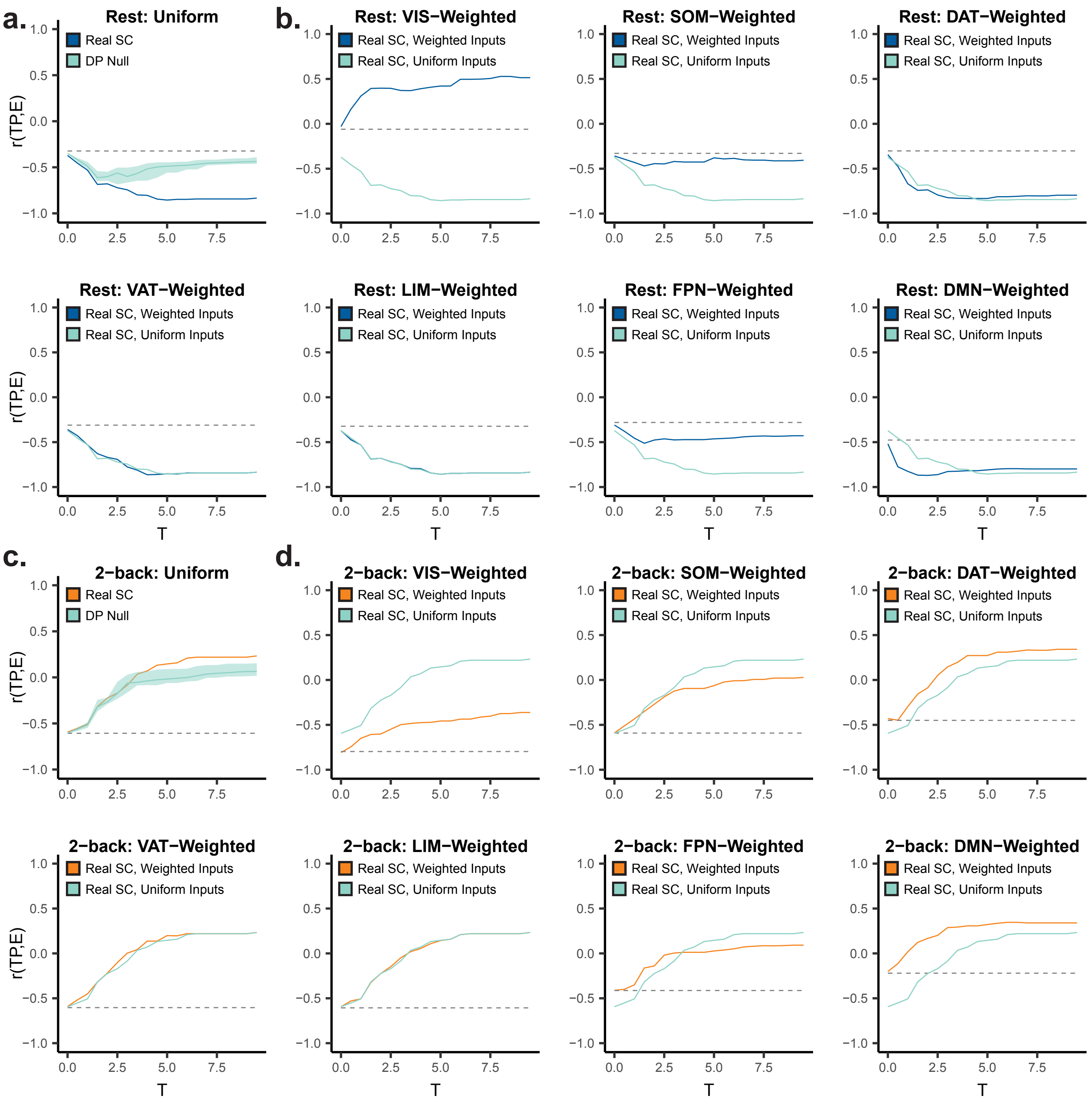

SUPPLEMENTARY FIGURE 11. **Selection of control horizon T and weighted control.** *(a-d)* Relationship between control horizon T (x-axis) and Spearman rank correlation (y-axis) between structurally predicted transition energies and empirically observed transition probabilities for resting state data (panel *a-b*) and 2-back task data (panel *c-d*). *(a, c)* Transition energies are computed for uniformly weighted inputs. Dashed line represents correlation between transition probabilities and $||\mathbf{x}_T - \mathbf{x}_o||$. Teal trace represents Spearman correlation using transition energies obtained from a distribution of null networks with preserved degree sequence (DP Null). Shaded region represents the full range of this distribution. *(b, d)* Transition energies are computed by weighting inputs towards different cognitive systems[1]. Dashed line represents correlation between transition probabilities and weighted state-space transition distance, computed as $(\mathbf{x}_T - \mathbf{x}_o)^\intercal(\mathbf{B}_\mathcal{K}\mathbf{B}_\mathcal{K}^\intercal)^{-1}(\mathbf{x}_T - \mathbf{x}_o)$. Teal trace represents Spearman correlation using transition energies computed with uniformly weighted inputs, the exact same as the blue and orange traces in panel *a* and panel *c*, respectively. *TP*, transition probability. *E*, minimum control energy $\mathbf{E}_m$.

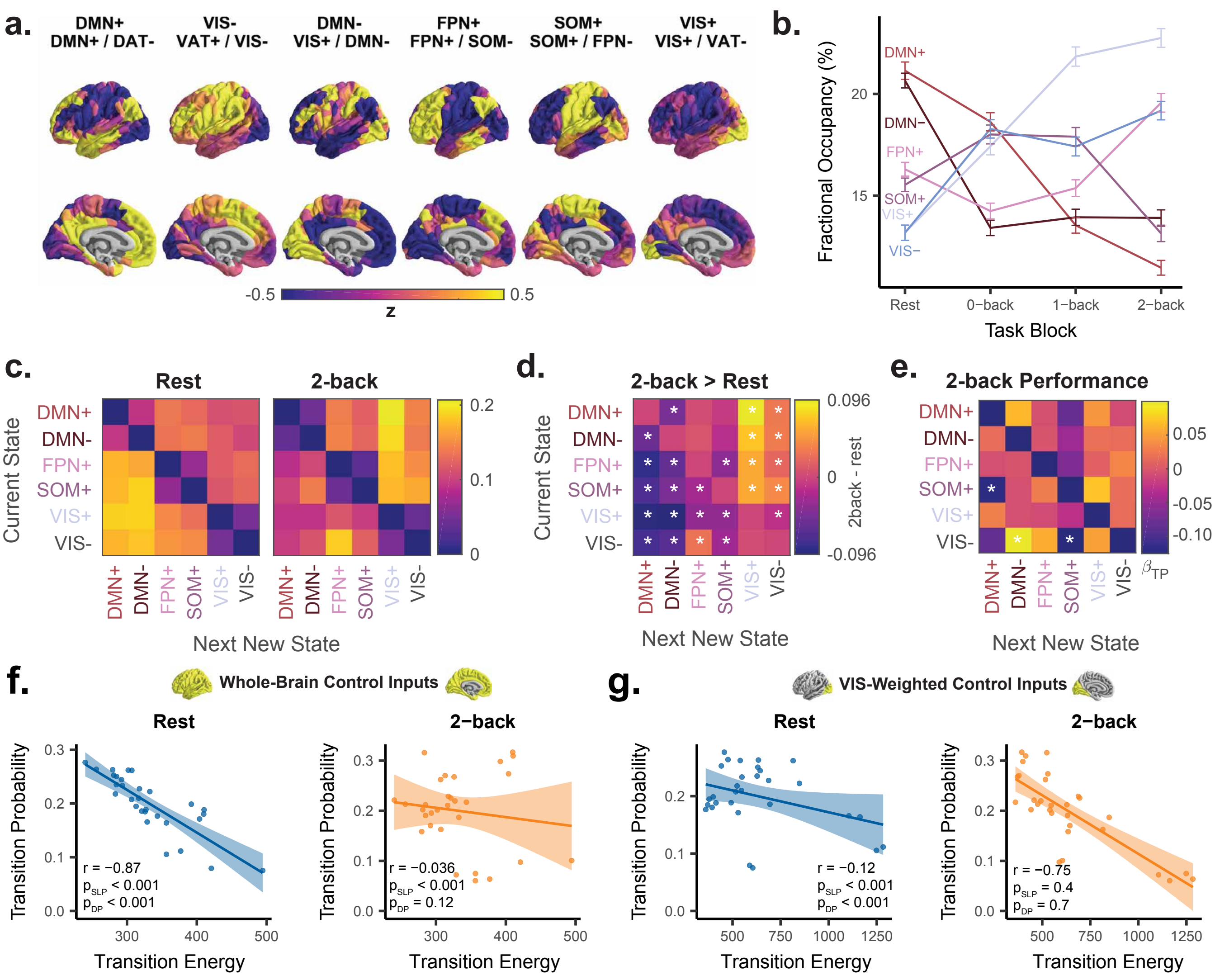

SUPPLEMENTARY FIGURE 12. **Key findings reproduced at** $k = 6$**.** *(a)* Cluster centroids at $k = 6$, similar to $k = 5$ with the addition of a SOM+ cluster. *(b)* State fractional occupancies change with increasing cognitive load similarly compared to $k = 5$. *(c-d)* Group average state transition probability matrices for rest (panel *(c)*) and 2-back condition of the n-back task (panel *(d)*) scans. *(e)* Permutation testing to compare 2-back and rest transition probabilities. *, statistically significant after Bonferroni correction over 30 transitions. *(f-g)* Correlation between structure-based transition energy prediction (x-axis) and empirically derived transition probability (y-axis) for resting state (left) and the 2-back condition of the n-back task (right), using inputs weighted evenly throughout the whole brain *(f)* or weighted positively towards the visual system *(g)*. *TP*, transition probability.

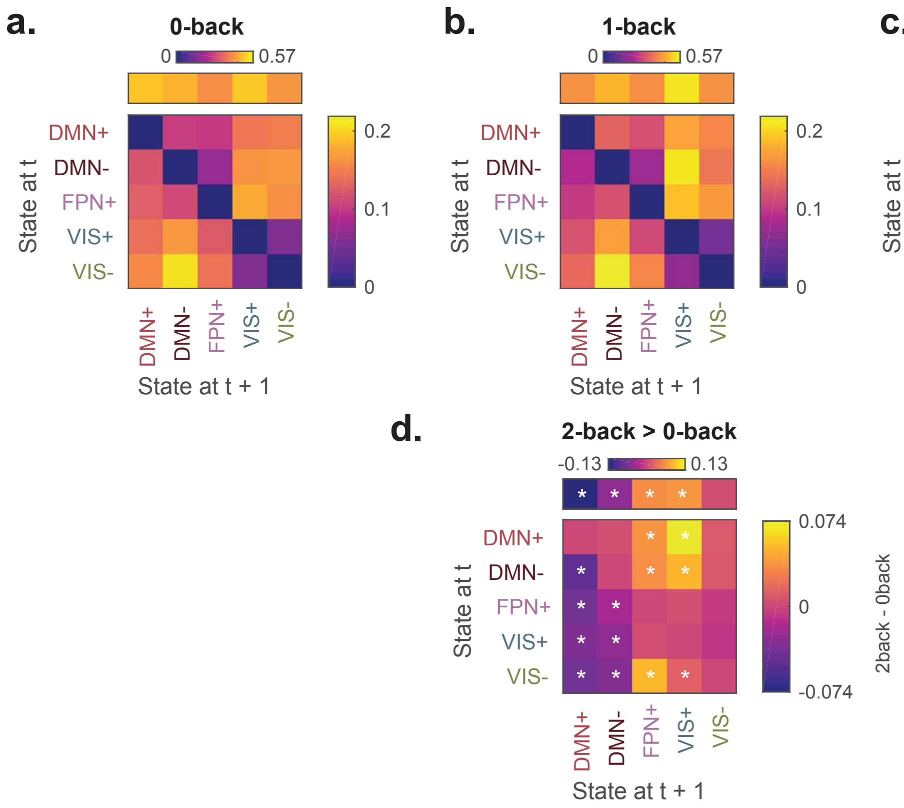

SUPPLEMENTARY FIGURE 13. **Brain state transitions within task blocks.** *(a-c)* Group average state transition probability matrices for 0-back (panel *a*), 1-back (panel *b*), and 2-back (panel *c*). Persistence probabilities are removed from the diagonal and depicted above the transition matrix. Heatmap color scale represents the probability of transitioning between two states from one BOLD frame to the next, or persisting in the same state. *(d)* Non-parametric permutation testing comparing transition probabilities in the 2-back to the 0-back block. *, two-tailed non-parametric $p_{\text{corr}} < 0.05$, after Bonferroni correction over 20 transition probabilities and 5 persistence probabilities.

Supplementary Notes, Discussion, and Methods

Sample exclusion criteria

We excluded 722 of the initial 1601 subjects for the following reasons: medical problems that may impact brain function, incidental radiologic abnormalities in brain structure, poor or incomplete FreeSurfer reconstruction of T1 images[5], high motion in rest or n-back fMRI scans, high signal-to-noise ratio or poor coverage in task-free or n-back task BOLD images, and failure to meet a rigorous manual and automated quality assurance protocol for DTI[6]. Notably, our goal in constructing a sample was to compare structure-function relationships between contexts across all subjects in our sample. This analysis required highly stringent inclusion criteria that only included subjects with high quality data for rest BOLD, n-back task BOLD, and DTI.

Functional Scan Types

During the resting-state scan, a fixation cross was displayed as images were acquired. Subjects were instructed to stay awake, keep their eyes open, fixate on the displayed crosshair, and remain still. Total resting state scan duration was 6.2 minutes. As previously described[7], we used the fractal n-back task[8] to measure working memory function. The task was chosen because it is a reliable probe of the executive system and is not contaminated by lexical processing abilities that also evolve during adolescence[9,10]. The task involved the presentation of complex geometric figures (fractals) for 500 ms, followed by a fixed interstimulus interval of 2500 ms. This occurred under the following three conditions: 0-back, 1-back, and 2-back, inducing different levels of working memory load. In the 0-back condition, participants responded with a button press to a specified target fractal. For the 1-back condition, participants responded if the current fractal was identical to the previous one; in the 2-back condition, participants responded if the current fractal was identical to the item presented two trials previously. Each condition consisted of a 20-trial block (60 s); each level was repeated over three blocks. The target/foil ratio was 1:3 in all blocks, with 45 targets and 135 foils overall. Visual instructions (9 s) preceded each block, informing the participant of the upcoming condition. The task included a total of 72 s of rest, while a fixation crosshair was displayed, which was distributed equally in three blocks of 24 s at the beginning, middle, and end of the task. Total task duration was 693 s. To assess performance on the task, we used d', a composite measure that takes into account both correct responses and false positives to separate performance from response bias[11].

Imaging data acquisition and preprocessing

MRI data were acquired on a 3 Tesla Siemens Tim Trio whole-body scanner and 32-channel head coil at the Hospital of the University of Pennsylvania. High-resolution T1-weighted images were acquired for each subject. For diffusion tensor imaging (DTI), 64 independent diffusion-weighted directions with a total of 7 $b = 0$ s/mm^2 acquisitions were obtained over two scanning sessions to enhance reliability in structural connectivity estimates[12]. All subjects underwent functional imaging (TR = 3000 ms; TE = 32 ms; flip angle = 90 degrees; FOV = 192×192 mm; matrix = 64×64; slices = 46; slice thickness = 3 mm; slice gap = 0 mm; effective voxel resolution = $3.0 \times 3.0 \times 3.0$ mm) during the resting-state sequence and the n-back task sequence[12,13]. During resting-state and n-back task imaging sequences, subjects' heads were stabilized in the head coil using one foam pad over each ear and a third pad over the top of the head in order to minimize motion. Prior to any image acquisition, subjects were acclimated to the MRI environment via a mock scanning session in a decommissioned scanner. Mock scanning was accompanied by acoustic recordings of gradient coil noise produced by each scanning pulse sequence. During these sessions, feedback regarding head motion was provided using the MoTrack motion tracking system (Psychology Software Tools, Inc., Sharpsburg, PA).

Raw resting-state and n-back task fMRI BOLD data were preprocessed following the most stringent of current standards[14,15] using XCP engine[16]: (1) distortion correction using FSL's FUGUE utility, (2) removal of the first 4 volumes of each acquisition, (3) template registration using MCFLIRT[17], (4) de-spiking using AFNI's 3DDESPIKE utility, (5) demeaning to remove linear or quadratic trends, (6) boundary-based registration to the individual high-resolution structural image, (7) 36-parameter global confound regression including framewise motion estimates and signal from white matter and cerebrospinal fluid, and (8) first-order Butterworth filtering to retain signal in the 0.01 to 0.08 Hz range. Following these preprocessing steps, we parcellated the voxel-level data using the 463-node Lausanne atlas[18]. We excluded the brainstem, leaving 462 parcels. Our choice of parcellation scale was motivated by prior work showing that parcellations of this scale replicate voxelwise clustering results more than coarser scales with fewer parcels[19]. We excluded any subject with mean relative framewise displacement > 0.5 mm or maximum displacement > 6 mm during the n-back scan, and mean relative framewise displacement > 0.2 mm for the resting state scan.

All DTI datasets were subject to a rigorous manual quality assessment protocol that has been previously described[6]. The skull was removed by applying a mask registered to a standard fractional anisotropy map (FMRIB58) to each subject's DTI image using an affine transformation. The FSL EDDY tool was used to correct

for eddy currents and subject motion and rotate diffusion gradient vectors accordingly. Distortion correction was applied using FSL's FUGUE utility. DSI studio was then used to estimate the diffusion tensor and perform deterministic whole-brain fiber tracking with a modified FACT algorithm that used exactly 1,000,000 streamlines per subject excluding streamlines with length < 10 mm[20,21]. Lausanne 463-node atlas parcels were extended into white matter with a 4 mm dilation[20,21] and then registered to the first $b = 0$ volume using an affine transform[6,20]. For all analyses, edge weights in the structural network were defined by the average fractional anisotropy value for streamlines connecting each pair of parcels[22].

Split-halves validation of clustering

To ensure that our final clustering solution for $k = 5$ was not influenced by outliers or adversely impacted by model overfitting, we split our sample into two equal partitions 500 times and performed k-means clustering separately on each half of the dataset. We then matched clusters by computing the cross-correlation between both sets of centroids, and then by reordering the clusters based on the maximum correlation value for each cluster. We plotted those maximum correlation values and found that most were > 0.99, suggesting a high degree of robustness and stability in brain states (Supplementary Fig. 2d). We also computed the state transition probabilities and state persistence probabilities for each half separately for rest and n-back, and then computed the correlation between the transition or persistence probabilities between the two data set partitions. Similarly, we found very high correlation values (> 0.99) for state transition probabilities and state persistence probabilities for both rest and n-back (Fig 2e-f). These observations suggest that our estimates of brain dynamics are robust to outliers and consistent across different subsamples of our data.

Functional connectivity does not fully explain instantaneous coactivation

We posit that the alignment between *a priori* resting state functional networks[1] (RSNs) and our coactivation patterns cannot be fully explained by analyzing interregional correlations in BOLD signal across time, which is often called "functional connectivity." Here, we provide evidence that suggests that our coactivation patterns are consistent with but not trivially explained by functional connectivity. We also provide examples that explain why temporal correlation does not necessarily explain instantaneous coactivation in general, and demonstrate that k-means clustering is a useful tool for extracting coactivation patterns.

In general, time series data can contain (1) unexpected coactivation patterns in the presence of temporal correlation and (2) unexpected coactivation patterns in the absence of temporal correlation. Here, we provide two examples that illustrate how k-means clustering can resolve dissociation between temporal correlation and instantaneous coactivation in multi-dimensional time series data. First, we show two stationary signals that are anticorrelated across time (Supplementary Fig. 1b, $r = -0.70$), but exhibit three unexpected coactivation patterns (rather than two simple "on-off" and "off-on" patterns) due to fluctuation between variable amplitudes (Supplementary Fig. 1a-b). In this example, one sinusoidal signal fluctuates between three distinct amplitudes and the other sinusoidal signal simply fluctuates between a peak and a trough (Supplementary Fig. 1a). The combination of these signals yields 3 distinct coactivation patterns, rather than the 2 patterns that would be expected from a pair of temporally anticorrelated signals (Supplementary Fig. 1c-e).

Next, we show two signals that are uncorrelated across time, but still have multiple spontaneous coactivation patterns (Supplementary Fig. 1a). Because these two signals sometimes activate together and at other times exhibit opposing activity, their temporal correlation is near 0 ($r = 0.018$), yet there are 3 unique coactivation patterns in the time series that k-means faithfully extracts. Positive temporal correlations could occur either through simultaneous activation or simultaneous inactivation, while temporal anticorrelation can result from opposing activity patterns. One could envision how adding in additional signals (i.e. dimensions or brain regions) could provide additional degrees of freedom to construct a range of coactivation patterns for a given temporal correlation structure. The sensitivity of the k-means approach to coactivation patterns in these two contexts supports its use in fMRI data for identifying previously unknown spontaneous interactions between neural networks, as well as the temporal organization of those networks.

When we apply k-means clustering to high-dimensional BOLD data from resting state and n-back scans (Fig. 2a), we identify activity patterns with both expected and unexpected features based on functional connectivity. In general, we saw that RSNs show coherent high or low amplitude activity in each state (Supplementary Fig. 8c). This finding reflects the strong positive correlations *within* RSNs (Supplementary Fig. 8a). We also see coactivation patterns consistent with patterns of functional connectivity *between* RSNs. For instance, the DMN+ state (Fig. 2a) shows spatial anticorrelation between the temporally anticorrelated dorsal attention network and default mode network[23] (Supplementary Fig. 8a, mean $r = -0.10$, one-sample t-test, $df = 878$, $t = -80.45$, $p < 10^{-15}$). However, we also identify coactivation patterns that do not trivially reflect functional connectivity. The DMN- state centroid (Fig. 2a) consists of low amplitude activity throughout the default mode network (DMN) with high amplitude activity throughout somatomotor and visual systems (Fig. 2c). When clustering

on functional connectivity[1], these three systems emerge as separate. Mean correlations within these systems are positive (Supplementary Fig. 8a-b), but correlations between them are near zero (SOM and VIS) or weakly negative (DMN with SOM and VIS) (Supplementary Fig. 8c). Based solely on functional connectivity, one would not expect these three systems to strongly coactivate or oppose one another at the level of individual BOLD frames (TRs), yet our analysis suggests that there are many TRs with low DMN activity, high VIS activity, and high SOM activity (Supplementary Fig. 8d-g). Moreover, VIS and SOM have two additional configurations of coactivation represented in the VIS+ and VIS- states (Fig. 2a-c). In the VIS- state, we see high amplitude SOM activity with low amplitude VIS activity, suggesting that at times VIS and SOM systems coactivate and at other times they oppose one another. In the VIS+ state we see low amplitude SOM activity with high amplitude VIS activity. Purely based on functional connectivity, one might inappropriately draw the conclusion that these two RSNs are independent given their mean correlation of zero, when in reality they have 3 distinct configurations at the level of individual time points. The behavior of VIS and SOM is most consistent with the scenario presented in Supplementary Fig. 1. Overall, these findings suggest that our analysis identifies recurrent activity patterns whose spatial organization reflects strong temporal correlations within RSNs, but also with coactivation between RSNs that cannot be trivially explained by temporal correlations between RSNs.

Silhouette analysis of clustering

In order to support the use of a discrete model of brain dynamics, we asked whether individual BOLD time points from rest and n-back scans exhibited clustering in a 462-region activation space. We used the silhouette scores of BOLD time points as a measure of clustering in this space. Silhouette scores range from -1 to 1 and are computed for each data point, with 1 indicating that a data point is closer to members of its assigned cluster than to members of the next closest cluster, 0 indicating equidistance between the assigned cluster and the closest cluster, and -1 indicating that the data point is closer to another cluster. We compared the silhouette scores for real BOLD data points from the PNC, data points from 462 independent random Gaussian distributions, and data points from independent phase randomized null time series[24] based on subject-specific BOLD data. This independent phase randomized null model (IPR) preserves the autocorrelation within each region, but destroys covariance between regions. When we compared the silhouette scores for clustering of real BOLD data to independent random Gaussian and autocorrelation-preserving null data, we found that the real data had higher mean silhouette scores than that of the autocorrelation-preserving null data (Supplementary Fig. 3a, difference in mean silhouette score actual minus null = 0.047, two sample t-test, $df = 27598$, $t = 115.03$, $p < 10^{-15}$). Additionally, the centroids generated by clustering this null data had little obvious structure (Supplementary Fig. 3e) and showed little similarity to the original centroids (Supplementary Fig. 3f). These findings suggest that BOLD data exhibit non-trivial clustering in regional activation space.

Impact of scan composition on brain states and dynamics

To ensure that our results were not biased by the fact that there were a larger number of n-back volumes (225 per scan) than rest volumes (120 per scan), we used the partition generated by clustering both entire scans together to compute separate centroids for volumes in rest or n-back scans. This analysis revealed a mean spatial Pearson correlation of 0.96 between corresponding centroids (Supplementary Fig. 4b). Next, we generated a new sample by concatenating the first 6 minutes of the n-back task data for each subject and the entire 6 minutes of the rest data for each subject. We ran this sample through the clustering algorithm at $k = 5$ and found that the cluster centroids (Supplementary Fig. 4c) were highly similar to those computed from the full sample (mean Pearson $r = 0.99$; Supplementary Fig. 4d). We also computed transition probabilities using this cluster partition and identified highly similar group average transition matrix structure (rest, Pearson $r = 0.997$, n-back, Pearson $r = 0.989$, Supplementary Fig. 4e), suggesting that the temporal order of state labels was largely unaffected by the scan composition of the sample. Moreover, these results suggest that n-back state transitions are internally consistent.

Finally, we show that the differences between rest and n-back in the proportion of subjects with any absent states (Supplementary Fig. 4f) is attenuated relative to the full sample (Supplementary Fig. 2e). This finding suggests that in the full sample, the n-back task data has better state representation due to better sampling, rather than poor classification of rest volumes. However, even with equal samples, the rest dataset still has more subjects with missing states, suggesting that there may be more variability in brain dynamics during rest. Importantly, there were still no subjects with absent states for rest or n-back at $k < 5$ (Supplementary Fig. 4f), and there was at least 1 subject with a missing state in rest or n-back at $k > 5$ (though bars are very small in Supplementary Fig. 4f). Collectively, these results support the simultaneous generation of partitions for rest and n-back volumes and the choice of $k = 5$ for analysis in the main text.

Impact of sampling rate, global signal regression, and head motion on brain states and dynamics

The BOLD data from the PNC was acquired at a sampling rate of one volume every 3 seconds[12], which is relatively slow compared with other large data sets, including the Human Connectome Project[25], which samples every 0.72 seconds. The standard preprocessing pipeline for this data set involves regression of head motion parameters, white matter confounds, cerebrospinal fluid confounds, and global signal from each voxel's time series[16,26]. It is controversial whether this procedure, known as "global signal regression," induces anticorrelation[27,28].

Thus, we selected 100 unrelated subjects from the minimally preprocessed version of the Human Connectome Project (HCP) data set[29] and performed the following preprocessing steps on resting state and n-back working memory task scans: (1) head motion regression, (2) linear and quadratic detrending, (3) bandpass filtering to retain the 0.01 to 0.08 Hz range, and (4) parcellation according to the 462 region Lausanne atlas. We concatenated all 405 volumes from the working memory task with the first 405 volumes from the resting state over all 100 subjects. We chose to make the number of volumes from each scan equal so that the clustering algorithm would not be biased towards one scan or the other.

Next, we performed k-means clustering on this matrix and computed the centroids (Supplementary Fig. 6a). Every HCP centroid was maximally correlated with only one PNC centroid, and *vice versa*, allowing for unambiguous matching between the two sets of brain states (Supplementary Fig. 6b). The DMN+ and DMN- states were the most similar to PNC states, while VIS+ and VIS- exhibited slightly lower correlations (Supplementary Fig. 6b). DMN+ and DMN- states, as well as VIS+ and VIS- states, exhibited strong anticorrelation with each other (Supplementary Fig. 6c). Nevertheless, the HCP off-diagonal elements of the transition probability matrices (i.e. transitions not persistence) for rest and 2-back block of the n-back task were correlated with PNC transition probabilities at $r = 0.74$ and $r = 0.57$, respectively (Supplementary Fig. 6d-e). This finding suggests that while there were differences in the spatial activity patterns of brain states, their dynamic progression through time was relatively similar. The unexplained variance between the two samples could be due to differences in age, with the PNC comprised of developing youths and the HCP comprised of healthy adults. Notably, task dynamics were less similar between the two groups, possibly reflecting stronger age-related changes in task dynamics relative to resting state. Moreover, the differences in transition probabilities between 2-back and rest were highly similar in HCP and in PNC (Supplementary Fig. 6e), with increased transitions from DMN and FPN states into VIS states. Overall, these findings suggest that while global signal regression and sampling rate may impact the spatial activity patterns comprising brain states to some degree, it does not impact estimation of their dynamics or the presence of spatial anticorrelation in their activity patterns.

Finally, we tested whether the identification of recurrent coactivation patterns with k-means clustering was biased by the inclusion of single frames with submillimeter framewise displacement. We removed 76,339 frames associated with > 0.1 mm framewise displacement, and repeated the clustering on the remaining 226,916 frames using correlation distance and $k = 5$. The resulting centroids (Supplementary Fig. 7a) were nearly identical to the centroids found in Figure 2a (Supplementary Fig. 7b, all $r > 0.99$ for corresponding centroids). These findings suggest that high motion frames minimally impact the clustering process. Therefore, we included these frames so that we could have the largest continuous sample of sequential frames from which to compute transition probabilities and dwell times.

Assessing randomness, asymmetry, autocorrelation, and distance dependence of brain state sequences

In addition to assessing the relationship between brain state transitions, cognitive demands, and behavior, we were also interested to assess important basic properties of the state transition probability matrix. First, we were interested to validate previous findings which suggest that the brain does not undergo every possible transition with equal probability[30]. For these analyses, we begin with a transition matrix (Supplementary Fig. 9a-b) whose ij^{th} element indicates the probability that state i occurs at time t and state j occurs at time $t + t_r$, where t_r is the repetition time (TR) of the scan (here, 3 seconds). The state transition probability matrix houses several pieces of important information. We refer to the diagonal entries in the transition probability matrix as the persistence probabilities, because they indicate the probability of remaining in a given state, and we refer to the off-diagonal entries in the transition probability matrix as the transition probabilities, because they indicate the probability of transitioning between two distinct states. Given this structure, we were interested to determine whether the brain dynamics that we observed could occur in a uniformly random distribution of states and state transitions. To test the randomness of persistence probabilities, we generated subject-level null state time series that preserved fractional occupancy but shuffled the temporal sequence of states; for example, if the state time series was given by the vector [1 1 2 2 3 3], then we would permute the order of the entries in that vector uniformly at random, yielding a distribution of vectors with the same proportion of each state, i.e. [1 2 1 3 3 2]. By comparing the observed persistence probabilities to the persistence probabilities in this null model, we can test whether the observed persistence probabilities would be expected based solely on fractional occupancy. We averaged together all subjects to generate a distribution

of group average null persistence probabilities and compared them to the empirically observed group average persistence probabilities.

To test the randomness of transition probabilities, we generated null state time series that preserved only the states involved in transitions and reduced sequences of repeating states to a single state. We removed repeating states to control for the potentially independent effects of state persistence, which is equivalent to temporal autocorrelation, in estimating transition probabilities. For example, if the state time series was given by the vector $V = [112233]$, then we would reduce that original vector to the new vector $V_t = [123]$, and subsequently permute the new vector uniformly at random. Specifically, for subject $i = 1, .., ..N$, we reduce the state sequence vector V_i to a transition sequence vector V_{t_i} by eliminating repeating states, compute a transition matrix T_i, and average across all subjects to generate a group average transition matrix $T = \frac{1}{N}\sum_{i=1}^{N} T_i$ that excludes state persistence (i.e. diagonal entries of T_i are equal to 0). Next, we shuffle V_{t_i} uniformly at random to generate V_{n_i}, compute a transition matrix T_{n_i}, and average across all subjects to generate a group average null transition matrix $T_n = \frac{1}{N}\sum_{i=1}^{N} T_{n_i}$ that excludes state persistence. We generate a distribution of T_n by independently shuffling V_{t_i} for each subject many times and averaging them across subjects. Finally, we compare each element of T to the corresponding element in a distribution of T_n to compute a two-tailed, non-parametric p-value for each transition. These analyses demonstrated that almost all of the observed persistence and transition probabilities in both resting state and the n-back task were unexpected under these uniformly random null models (all $p_{\text{corr}} < 0.05$ except for DMN- to DMN+ during n-back, Supplementary Fig. 9a-b).

Next, we assessed the properties of these transition matrices, such as the matrix symmetry, which reflects whether transitions from state $1 \rightarrow 2$ occur as frequently as transitions from state $2 \rightarrow 1$, and so forth for every state pair. Specifically, we quantified the asymmetry ψ of a k-by-k transition matrix $\mathbf{A}$ as:

$$\psi = 0.5 \times \frac{\sum_{i=1}^{k}\sum_{j=1,j\neq i}^{k} |\mathbf{A}_{ij} - \mathbf{A}^{\intercal}_{ij}|}{\sum_{i=1}^{k}\sum_{j=1,j\neq i}^{k} |\mathbf{A}_{ij}|} .$$

In calculating this asymmetry score, we exclude the elements along the diagonal of $\mathbf{A}$ so as to only capture directional bias in transitions between pairs of states, without including the probability of persisting in each state. The values of this score range from 0 to 1, where 0 represents a matrix that is symmetric about the diagonal and 1 represents a matrix in which the upper triangle is $-1\times$ the lower triangle.

We were also interested in how much information about future states was contained within the current state. Drawing from information theoretic approaches to analysis of discrete signals, we computed the normalized auto mutual information (NMI) between lagged state time series to answer this question. Here, we asked whether the current state contains information about the subsequent state by computing NMI between the original state time series and a state time series lagged by one element. First, we created two copies of the state time series. Then, we removed the first element from one, $\mathbf{X}$, and the last element from the other, $\mathbf{Y}$, to generate two vectors of equal length such that $\mathbf{X}_i = \mathbf{Y}_{i+1}$. We computed the NMI between $\mathbf{X}$ and $\mathbf{Y}$ as:

$$\frac{\mathbf{H}(\mathbf{X}) - \mathbf{H}(\mathbf{X}|\mathbf{Y})}{\mathbf{H}(\mathbf{X})} ,$$

where $\mathbf{H}(\mathbf{X})$ is the entropy of $\mathbf{X}$ and $\mathbf{H}(\mathbf{X}|\mathbf{Y})$ is the conditional entropy of $\mathbf{X}$ given $\mathbf{Y}$. Where k is the number of brain states,

$$\mathbf{H}(\mathbf{X}) = \sum_{i=1}^{k} P(\mathbf{X} = i) \times log(P(\mathbf{X} = i)) ,$$

and

$$\mathbf{H}(\mathbf{X}|\mathbf{Y}) = \sum_{i=1}^{k}\sum_{j=1}^{k} P(\mathbf{X} = i \wedge \mathbf{Y} = j) \times log(P(\mathbf{X} = i \wedge \mathbf{Y} = j)) .$$

In normalizing by $\mathbf{H}(\mathbf{X})$, we ensure that the NMI ranges from 0 to 1, with 0 representing two completely independent signals and 1 representing two identical signals.

We anticipated that stimulus driven activity in the n-back task that occurs independently of the current brain state would result in a reduction of directional, asymmetric state transitions relative to resting state and a reduction in the dependency of state transitions on the current state. Indeed, using the normalized measure of matrix skewness described above, we found that transition probabilities at rest were more asymmetric than during the n-back task (Supplementary Fig. 9d; paired t-test, $\mu_{nback-rest} = -0.062$, $df = 878$, $t = -24.96$, $p < 10^{-15}$)s. Additionally, using the normalized auto-mutual information metric described above, we found that the current brain state carried less information about the subsequent state during the n-back task relative to rest, even when controlling for autocorrelation (Supplementary Fig. 9f: normalized auto mutual information, $\mu_{nback-rest} = -0.028$, $df = 878$, $t = -20.88$, $p_{\text{corr}} < 10^{-15}$). Consistent with our hypothesis, we also found that the Euclidean distance between states was anticorrelated with the transition probabilities between states (Supplementary Fig. 9e). Interestingly, however, the effect was stronger for n-back than for rest (Supplementary Fig. 9e; paired t-test, $\mu_{nback-rest} = -0.17$, $df = 878$, $t = -22.74$, $p < 10^{-15}$), suggesting that the brain is more prone to transition between distant states while at rest.

Transition probabilities within task blocks

In addition to computing transition probabilities across the entire n-back task scan, we also computed transition probabilities within each task block. Because the instances of a specific task block (i.e. 0-back, 1-back, 2-back) are not continuous, we counted the number of each transition found within all instances of a given task block, and then we divided the counts by the total number of possible transitions within all instances of that task block (Supplementary Fig. 13a-c). Similar to the analysis of transition probabilities in the entire n-back scan, we found that in the 2-back block, transitions from the DMN+ and DMN- states into the VIS+ state were increased relative to the 0-back block (Supplementary Fig. 13d). Interestingly, we saw that transitions from DMN+ and DMN- states in to FPN+ states increased from 0-back to 2-back (Supplementary Fig. 13d), although in the resting state these transitions were more frequent relative to the entire n-back scan (Fig. 4c). However, transitions from VIS+ to FPN+ did not differ between the two conditions, while transitions into DMN+ and DMN- states decreased (Supplementary Fig. 13d). These findings suggest that increasing cognitive load biases the traversal of some trajectories in state space without affecting others.

Spatially embedded null models

To assay the specificity of brain state activity patterns themselves, we compared $\mathbf{P}_e$ for actual brain states to $\mathbf{P}_e$ for a distribution of null brain states. We generated null states using a recent method developed to find overlap between activation maps while accounting for spatial clustering of activity[2]. Following this method[2], we projected node-level data to a cortical surface, inflated the surface to a sphere using FreeSurfer tools, applied a rotation to the sphere, collapsed it back to a cortical surface, and extracted node-level data by averaging over vertices belonging to each region. This process preserves the spatial grouping and relative locations of regions with similar activity while still changing their absolute locations. Importantly, reflected versions of the same rotation are applied for each hemisphere, thus also preserving the symmetry of the original activity pattern.

To assay the specificity of our findings to higher order topological features found in real structural brain networks, we compared $\mathbf{P}_e$ estimates to a recently developed network null model[4], which preserves several important spatial and topological features. This model exactly preserves the degree sequence and edge weight distribution, while approximately preserving the edge length distribution and edge length-weight relationship. We also compared our findings to a more commonly used topological null model[3] which preserves only the degree distribution, but not the degree sequence, of the network.

Comparing transition energies and transition probabilities using control theory

A main goal of the present work was to provide a mechanistic description for how the brain's large-scale white matter architecture constrains its progression through activation space. To accomplish this goal, we began with a simple model of linear, time-invariant dynamics along the white matter structural connectome estimated from diffusion tractography. We represent the volume-normalized, fractional anisotropy-weighted structural network as the graph $\mathcal{G} = (\mathcal{V}, \mathcal{E})$, where $\mathcal{V}$ and $\mathcal{E}$ are the vertex and edge sets, respectively. Let $\mathbf{A}_{ij}$ be the weight associated with the edge $(i, j) \in \mathcal{E}$, and define the weighted adjacency matrix of $\mathcal{G}$ as $\mathbf{A} = [\mathbf{A}_{ij}]$, where $\mathbf{A}_{ij} = 0$ whenever $(i, j) \notin \mathcal{E}$. We associate a real value with each of the N brain regions to generate a vector describing the activity in each node at time t, and we define the map $\mathbf{x} : \mathbb{R}_{\geq 0} \to \mathbb{R}^N$ to describe the dynamics of activity in network nodes over time. Here we employ a simplified noise-free linear continuous-time and time-invariant model of such dynamics:

$$\dot{\mathbf{x}}(t) = \mathbf{A}\mathbf{x}(t) + \mathbf{B}\mathbf{u}(t) \ , \tag{1}$$

where $\mathbf{x}$ describes the activity (i.e. voltage, firing rate, BOLD signal) of brain regions over time. Thus, the vector $\mathbf{x}$ has length N, where N is the number of brain regions in the parcellation, and the value of $\mathbf{x}_i$ describes the activity level of that region. The matrix $\mathbf{A}$ is symmetric, with the diagonal elements satisfying $\mathbf{A}_{ii} = 0$. Prior to calculating control energy, we divide $\mathbf{A}$ by $\xi_0(\mathbf{A})$ and subtract 1 from the diagonal elements of $\mathbf{A}$, where $\xi_0(\mathbf{A})$ is the largest eigenvalue of $\mathbf{A}$. This step makes the system marginally stable by ensuring that the maximum eigenvalue of the system is equal to 0. The input matrix $\mathbf{B}$ identifies the control input weights, which we set to the $N \times N$ identity matrix by default. For certain analyses (Fig. 5d, Supplementary Fig. 11b and d, Supplementary Fig. 5d, Supplementary Fig. 12d), a set of brain regions $\mathcal{K} \subset \mathcal{V}$ that belong to a particular cognitive system[1] was given increased weight, such that

$$\mathbf{B}_{ii} = \begin{cases} c & \text{if } i \in \mathcal{K} \\ 1 & \text{otherwise} \end{cases} , \tag{2}$$

and c is a positive, real scalar value, which we set equal to 2 here. The input $\mathbf{u} : \mathbb{R}_{\geq 0} \to \mathbb{R}^M$ denotes the control strategy.

To compute the minimum control energy required to drive the system from an initial activity pattern $\mathbf{x}_o$ to a final activity pattern $\mathbf{x}_f$ over some time T, we compute an invertible controllability Gramian $\mathbf{W}$ for controlling the network $\mathbf{A}$ from the set of network nodes $\mathcal{K}$ (in our case, every node in the network), where:

$$\mathbf{W} = \int_0^T e^{\mathbf{A}(T-\tau)}\mathbf{B}\mathbf{B}^\intercal e^{\mathbf{A}^\intercal(T-\tau)}d\tau, \tag{3}$$

where T is the time horizon, which specifies the time over which input to the system is allowed. After computing the controllability Gramian, we can solve for the minimum control energy $\mathbf{E_m}$ by computing the quadratic product between the inverted controllability Gramian and the difference between $\mathbf{x}_o$ and $\mathbf{x}_T$:

$$\mathbf{E_m} = (e^{\mathbf{A}T}\mathbf{x_o} - \mathbf{x_T})^\intercal \mathbf{W}^{-1}(e^{\mathbf{A}T}\mathbf{x_o} - \mathbf{x_T})\ . \tag{4}$$

In Fig. 5b, we computed the $k \times k$ transition energy matrix $\mathbf{T}_e$ as the minimum energy required to transition between all possible pairs of the k clustered brain states, given the white matter connections represented in $\mathbf{A}$. We refer to the on-diagonal elements of $\mathbf{T}_e$ as *persistence energies*, because they quantify the energy required to maintain $\mathbf{x}_o$ in the special case where $\mathbf{x}_o = \mathbf{x}_T$. We refer to the off-diagonal elements of $\mathbf{T}_e$ as *transition energies*, because they quantify the energy required to move between all pairs of $\mathbf{x}_o$ and $\mathbf{x}_T$ where $\mathbf{x}_o \neq \mathbf{x}_T$.

First, we sought to determine whether the brain states that we observed (Fig. 2a) were easier to maintain (1) compared to null states and (2) in real brain networks compared to null brain networks. Thus, we compared the persistence energy for the actual states to that of null brain states[2] with preserved symmetry and spatial clustering (Supplementary Fig. 10a). We found that the persistence energy of the DMN+ state was significantly lower than that of its respective null states (Supplementary Fig. 10b: one-tailed non-parametric test, DMN+, $p_{\text{corr}} = 0.045$, 1000 sphere-permuted null states). The DMN- state also required lower persistence energy than many of its respective null states, although this result was not significant after Bonferroni correction over all states (Fig. 5b, one-tailed non-parametric test, $p_{\text{corr}} = 0.05$, 1000 sphere-permuted null states). Crucially, the DP and SLP null models did not exhibit selectively increased stability in DMN states, suggesting that DMN-driven states may arise in part due to complex features of white matter topology that allow for their stability.

Next, we hypothesized that the off-diagonal elements of the empirically observed state transition matrix at rest (Fig. 4a) would be anticorrelated with the off-diagonal elements of $\mathbf{T}_e$. This hypothesis was based on the notion that the brain would empirically prefer trajectories in state space requiring smaller magnitude control inputs to achieve. Note that we ignored the diagonal of $\mathbf{T}_e$, which captures whether persistence energies explain observed state dwell times, a question that we were not sufficiently powered to ask with only $k = 5$ states but that should be revisited in future studies.

The choice of the control horizon T is critical for the calculation of $\mathbf{T}_e$. When control inputs are uniformly distributed across the brain such that $\mathbf{B}_\mathcal{K}$ is the identity matrix, then as T approaches 0, the minimum control energy $\mathbf{E}_m$ becomes proportional to the squared Euclidean norm of $\mathbf{x}_T - \mathbf{x}_o$. $||\mathbf{x}_T - \mathbf{x}_o||^2$ is the state space distance between the initial and final state in the transition. At longer time horizons, both $||\mathbf{x}_T - \mathbf{x}_o||^2$ and the topology of $\mathbf{A}$ determine $\mathbf{E}_m$. When $\mathbf{B}_\mathcal{K}$ is not the identity matrix, $\mathbf{E}_m$ becomes proportional to $(\mathbf{x}_T - \mathbf{x}_o)^\intercal(\mathbf{B}_\mathcal{K}\mathbf{B}_\mathcal{K}^\intercal)^{-1}(\mathbf{x}_T - \mathbf{x}_o)$ as T approaches 0.

However, because the units of the edge weights of $\mathbf{A}$, obtained from deterministic tractography performed on diffusion-weighted imaging data, are not measured in activity per unit time, the value of T is arbitrary relative to $\mathbf{A}$ and the real time in which neural activity was measured through BOLD fMRI. Therefore, we chose T in a data-driven fashion by computing transition energies for a range of T values using a group representative structural $\mathbf{A}$ matrix, and computing the Spearman rank correlation between transition energies and the group average transition probability matrix from resting state fMRI data (Supplementary Fig. 11a). We used the Spearman rank correlation rather than the Pearson correlation in order to reduce the effect of outliers on estimating the relationship between transition probability and transition energy. We found that the strongest negative correlation value was obtained for $T = 5$, but similarly strong negative correlations were found for the range $T = [5, 10]$ (Supplementary Fig. 11a). Accordingly, we used $T = 5$ for the analyses presented in Fig. 5b-c, Fig. 6c, and Supplementary Fig. 10b. We also carried out the same analysis using a distribution of null networks with preserved degree sequence (see Fig. 5b), which revealed that there was no T value yielding a correlation between transition energies and observed resting state transition probabilities as strongly negative as when we used the real structural connectivity matrix (Supplementary Fig. 11a).

In addition to controlling the brain with uniformly weighted inputs, we also asked whether transition energies obtained using a non-uniform distribution of inputs might better explain the observed transition probabilities. Specifically, we hypothesized that accounting for external visual input during the fractal n-back task might provide a more accurate estimation of the input energy needed to achieve each transition. We weighted the inputs towards one cognitive system[1] at a time while still allowing input into every brain region, and then recomputed the Spearman correlation between transition energies and transition probabilities for the resting state (Supplementary Fig. 11b) and the 2-back condition of the n-back task (Supplementary Fig. 11d). This analysis revealed that accounting for visual input in computing transition energies improved our ability to explain the observed brain state transition probabilities during the 2-back condition (Supplementary Fig. 11d VIS-weighted subpanel, orange trace is lower than teal trace), and abolished our ability to explain resting state transition probabilities (Supplementary Fig. 11b VIS-weighted subpanel, blue trace is greater than 0 and dashed line is near 0). However, we did not find a clear role for brain structure in this relationship, as evidenced by the fact

that transition energy did not explain transition probability any better than the weighted distance between states (Supplementary Fig. 11d, orange trace does not dip below dashed line). Thus, we present results in Fig. 5d for $T = 0.001$, where $\mathbf{E}_m$ is proportional to $(\mathbf{x}_T - \mathbf{x}_o)^\intercal(\mathbf{B}_\mathcal{K}\mathbf{B}_\mathcal{K}^\intercal)^{-1}(\mathbf{x}_T - \mathbf{x}_o)$. Nevertheless, this finding suggests a specificity of the constraints of state-space transition distance on the brain's empirically observed progression through state space. Resting state transition probabilities can be explained by unweighted state-space distance (Supplementary Fig. 11a, dashed line, Spearman's $r = -0.32$) but not by visual system-weighted state-space distance (Supplementary Fig. 11b, VIS-weighted dashed line, Spearman's $r = -0.06$); 2-back transition probabilities can be explained by unweighted state-space distance (Supplementary Fig. 11c, dashed line, Spearman's $r = -0.61$), but are explained best by visual system-weighted state-space distance (Supplementary Fig. 11b, VIS-weighted dashed line, Spearman's $r = -0.80$). This result suggests that visual input allows the brain to deviate from the constraints of state-space distance found at rest, while an equal consideration of visual inputs alongside other inputs is key to explaining resting state transitions. Resolving the effect of structure on brain dynamics during a task may require full knowledge of all input sources, which could potentially be uncovered through a data-driven approach[31].

Impact of parcellation and cluster number

The choice of parcellation scale may impact analyses involving tractography, where relative region sizes may bias estimates of connectivity. We chose a relatively fine-grained parcellation with 462 nodes because previous coactivation pattern analyses[19] at this scale produced cluster assignments similar to those obtained by clustering at the voxel level. However, we were interested to know whether we would obtain the same results using a coarser parcellation scale. Therefore, we repeated the clustering procedure and subsequent control theoretic analyses using the Lausanne 234 node parcellation[18].

First, this analysis revealed brain states whose spatial maps were virtually identical to those generated using the 462 node parcellation (Supplementary Fig. 5a). The fractional occupancy of these states also changed with cognitive load in a similar fashion (Supplementary Fig. 5a) compared to the results in Fig. 3d. Similar to the results presented in the main text, we found a negative relationship between transition energies and empirically observed brain state transition probabilities at rest (Supplementary Fig. 5c, Spearman's $r = -0.62$, $p_{SLP} < 0.001$, $p_{DP} < 0.001$) with a weak positive relationship between transition energies and transition probabilities from the 2-back condition (Supplementary Fig. 5c, Spearman's $r = 0.21$, $p_{SLP} = 1$, $p_{DP} = 0.85$). We also found that discounting the weight of the visual system in calculating state-space transition distance allowed us to better explain 2-back transition probabilities (Supplementary Fig. 5d, Spearman's $r = -0.78$, $p_{SLP} = 1$, $p_{DP} = 1$), and reduced our ability to explain resting state transition probabilities (Supplementary Fig. 5d, Spearman's $r = -0.31$, $p_{SLP} < 0.001$, $p_{DP} < 0.001$). These results suggest that one can quantify the constraints of white matter architecture on brain state transitions at rest at multiple scales of region definition, supporting the generalizability of our findings.

A limitation of k-means clustering is the need to specify an absolute number of clusters. While the data suggests that $k = 5$ is the optimal solution, one could certainly choose to analyze this data at multiple scales. Because of a recent study[32] in which $k = 6$ was identified as the optimal solution by the same heuristic, we reproduce our results at $k = 6$ in the interest of facilitating comparison between the two studies.

At $k = 6$, cluster centroid brain states interestingly become grouped into 3 anticorrelated pairs (DMN+ and DMN-, VIS+ and VIS-, FPN+ and SOM+), similar to Ref.[32]. The states were also highly similar to those at $k = 5$, with the DMN- state from $k = 5$ essentially "split" into the SOM+ and DMN- state at $k = 6$. The DMN- state at $k = 6$ state houses concurrent high amplitude activity in the dorsal attention network and visual system with low amplitude activity in the default mode network (Supplementary Fig. 12a). This appearance of "split" brain states is consistent with hierarchical state organization[30,33], which becomes apparent at different clustering scales. As WM load increased from 0-back to 2-back, we saw a decrease in DMN+ fractional occupancies with a concurrent increase in FPN+ and VIS state fractional occupancies (Supplementary Fig. 12b). Direct transitions between anticorrelated states were infrequent (Supplementary Fig. 12c-d), with a drastic shift in transition probabilities towards VIS states and away from DMN states during 2-back relative to rest (Supplementary Fig. 12e). Similar to $k = 5$, at $k = 6$ we found that 2-back task performance was related to 2-back state transitions from the VIS- state into states with coherent frontoparietal and default mode activity (Supplementary Fig. 12e). Specifically, we found that transitions from VIS- into DMN- and FPN+ were positively associated with performance, consistent with the high amplitude dorsal attention network activity and low amplitude DMN activity found in the DMN- state (Supplementary Fig. 12a). Additionally, transitions from the VIS- state to the SOM+ state were negatively associated with performance, consistent with the frontoparietal deactivation found in the SOM+ state (Supplementary Fig. 12a). We again found a strong negative correlation between transition energies and resting state transition probabilities (Supplementary Fig. 12f, Spearman's $r = -0.87$, $p_{SLP} < 0.001$, $p_{DP} < 0.001$) that was specific to the topology of white matter, supporting the notion that the constraints of white matter on state-space progression generalizes across multiple scales of states. Similar to our results at $k = 5$, we found that discount-

ing the weight of the visual system in calculating the state-space distance of transitions allowed us to better explain the state transitions observed during the 2-back task, during which visual stimuli are frequently delivered (Supplementary Fig. 12g, Spearman's $r = -0.75$, $p_{SLP} = 0.4$, $p_{DP} = 0.7$). Overall, this analysis suggests that our main findings hold true at near optimal values of k, and that much can be learned from studying the states of the brain at different scales.

---

[1] Yeo BT, Krienen FM, Sepulcre J, Sabuncu MR, Lashkari D, Hollinshead M, Roffman JL, Smoller JW, Zollei L, Polimeni JR *et al.*: **The organization of the human cerebral cortex estimated by intrinsic functional connectivity**. *Journal of Neurophysiology* 2011. **106**:1125–1165. doi:10.1152/jn.00338.2011.

[2] Alexander-Bloch A, Shou H, Liu S, Satterthwaite TD, Glahn DC, Shinohara RT, Vandekar SN, and Raznahan A: **On testing for spatial correspondence between maps of human brain structure and function**. *NeuroImage* 2018. **178**:540–551. doi: 10.1016/j.neuroimage.2018.05.070.

[3] Rubinov M and Sporns O: **Complex network measures of brain connectivity: Uses and interpretations**. *NeuroImage* 2010. **52**:1059–1069. doi: 10.1016/J.NEUROIMAGE.2009.10.003.

[4] Betzel RF and Bassett DS: **The specificity and robustness of long-distance connections in weighted, interareal connectomes**. *Proceedings of the National Academy of Sciences of the United States of America* 2017. **115**:E4880–E4889. doi: 10.1073/pnas.1720186115.

[5] Rosen AF, Roalf DR, Ruparel K, Blake J, Seelaus K, Villa LP, Ciric R, Cook PA, Davatzikos C, Elliott MA *et al.*: **Quantitative assessment of structural image quality**. *NeuroImage* 2018. **169**:407–418. doi: 10.1016/j.neuroimage.2017.12.059.

[6] Roalf DR, Quarmley M, Elliott MA, Satterthwaite TD, Vandekar SN, Ruparel K, Gennatas ED, Calkins ME, Moore TM, Hopson R *et al.*: **The impact of quality assurance assessment on diffusion tensor imaging outcomes in a large-scale population-based cohort**. *NeuroImage* 2016. **125**:903–919. doi: 10.1016/j.neuroimage.2015.10.068.

[7] Satterthwaite TD, Ruparel K, Loughead J, Elliott MA, Gerraty RT, Calkins ME, Hakonarson H, Gur RC, Gur RE, and Wolf DH: **Being right is its own reward: Load and performance related ventral striatum activation to correct responses during a working memory task in youth**. *NeuroImage* 2012. **61**:723–729. doi:10.1016/j.neuroimage.2012.03.060.

[8] Ragland JD, Turetsky BI, Gur RC, Gunning-Dixon F, Turner T, Schroeder L, Chan R, and Gur RE: **Working memory for complex figures: an fMRI comparison of letter and fractal n-back tasks.** *Neuropsychology* 2002. **16**:370–9.

[9] Schlaggar BL, Brown TT, Lugar HM, Visscher KM, Miezin FM, and Petersen SE: **Functional Neuroanatomical Differences Between Adults and School-Age Children in the Processing of Single Words**. *Science* 2002. **296**:1476–1479. doi: 10.1126/science.1069464.

[10] Brown TT, Lugar HM, Coalson RS, Miezin FM, Petersen SE, and Schlaggar BL: **Developmental Changes in Human Cerebral Functional Organization for Word Generation**. *Cerebral Cortex* 2005. **15**:275–290. doi:10.1093/cercor/bhh129.

[11] Snodgrass JG and Corwin J: **Pragmatics of MEasuring Recogntion Memory: Application to Dementia and Amnesia**. *Journal of Experimental Psychology* 1988. **117**:34–50.

[12] Satterthwaite TD, Elliott MA, Ruparel K, Loughead J, Prabhakaran K, Calkins ME, Hopson R, Jackson C, Keefe J, Riley M *et al.*: **Neuroimaging of the Philadelphia Neurodevelopmental Cohort**. *NeuroImage* 2014. **86**:544–553. doi: 10.1016/j.neuroimage.2013.07.064.

[13] Satterthwaite TD, Wolf DH, Erus G, Ruparel K, Elliott MA, Gennatas ED, Hopson R, Jackson C, Prabhakaran K, Bilker WB *et al.*: **Functional Maturation of the Executive System during Adolescence**. *Journal of Neuroscience* 2013. **33**:16249–16261. doi: 10.1523/JNEUROSCI.2345-13.2013.

[14] Satterthwaite TD, Elliott MA, Gerraty RT, Ruparel K, Loughead J, Calkins ME, Eickhoff SB, Hakonarson H, Gur RC, Gur RE *et al.*: **An improved framework for confound regression and filtering for control of motion artifact in the preprocessing of resting-state functional connectivity data**. *NeuroImage* 2013. **64**:240–256. doi:10.1016/j.neuroimage.2012.08.052.

[15] Ciric R, Wolf DH, Power JD, Roalf DR, Baum GL, Ruparel K, Shinohara RT, Elliott MA, Eickhoff SB, Davatzikos C *et al.*: **Benchmarking of participant-level confound regression strategies for the control of motion artifact in studies of functional connectivity**. *NeuroImage* 2017. **154**:174–187. doi: 10.1016/j.neuroimage.2017.03.020.

[16] Ciric R, Rosen AFG, Erus G, Cieslak M, Adebimpe A, Cook PA, Bassett DS, Davatzikos C, Wolf DH, and Satterthwaite TD: **Mitigating head motion artifact in functional connectivity MRI**. *Nature Protocols* 2018. **13**:2801–2826. doi:10.1038/s41596-018-0065-y.

[17] Jenkinson M, Bannister P, Brady M, and Smith S: **Improved optimization for the robust and accurate linear registration and motion correction of brain images.** *NeuroImage* 2002. **17**:825–41.

[18] Cammoun L, Gigandet X, Meskaldji D, Thiran JP, Sporns O, Do KQ, Maeder P, Meuli R, and Hagmann P: **Mapping the human connectome at multiple scales with diffusion spectrum MRI**. *Journal of Neuroscience Methods* 2012. **203**:386–397. doi: 10.1016/j.jneumeth.2011.09.031.

[19] Chen JE, Chang C, Greicius MD, and Glover GH: **Introducing co-activation pattern metrics to quantify spontaneous brain network dynamics** 2015. doi: 10.1016/j.neuroimage.2015.01.057.

[20] Baum GL, Roalf DR, Cook PA, Ciric R, Rosen AFG, Xia C, Elliot MA, Ruparel K, Verma R, Tunc B *et al.*:

The Impact of In-Scanner Head Motion on Structural Connectivity Derived from Diffusion Tensor Imaging. *bioRxiv* 2017. 185397. doi:10.1101/185397.

[21] Gu S, Pasqualetti F, Cieslak M, Telesford QK, Yu AB, Kahn AE, Medaglia JD, Vettel JM, Miller MB, Grafton ST *et al.*: **Controllability of structural brain networks**. *Nature Communications* 2015. **6**. doi: 10.1038/ncomms9414.

[22] Baum GL, Ciric R, Roalf DR, Betzel RF, Moore TM, Shinohara RT, Kahn AE, Vandekar SN, Rupert PE, Quarmley M *et al.*: **Modular Segregation of Structural Brain Networks Supports the Development of Executive Function in Youth.** *Current biology : CB* 2017. **27**:1561–1572.e8. doi:10.1016/j.cub.2017.04.051.

[23] Fox MD, Snyder AZ, Vincent JL, Corbetta M, Van Essen DC, and Raichle ME: **From The Cover: The human brain is intrinsically organized into dynamic, anticorrelated functional networks**. *Proceedings of the National Academy of Sciences* 2005. **102**:9673–9678. doi: 10.1073/pnas.0504136102.

[24] Liégeois R, Laumann TO, Snyder AZ, Zhou J, and Yeo BT: **Interpreting temporal fluctuations in resting-state functional connectivity MRI**. *NeuroImage* 2017. **163**:437–455. doi: 10.1016/J.NEUROIMAGE.2017.09.012.

[25] Van Essen DC, Smith SM, Barch DM, Behrens TE, Yacoub E, and Ugurbil K: **The WU-Minn Human Connectome Project: An overview**. *NeuroImage* 2013. **80**:62–79. doi:10.1016/j.neuroimage.2013.05.041.

[26] Xia CH, Ma Z, Ciric R, Gu S, Betzel RF, Kaczkurkin AN, Calkin ME, Cook PA, Garcia de la Garza A, Vandekar S *et al.*: **Linked dimensions of psychopathology and connectivity in functional brain networks**. *DoiOrg* 2017. 199406. doi:10.1101/199406.

[27] Chai XJ, Castañán AN, Öngür D, and Whitfield-Gabrieli S: **Anticorrelations in resting state networks without global signal regression**. *NeuroImage* 2012. **59**:1420–1428. doi:10.1016/j.neuroimage.2011.08.048.

[28] Murphy K and Fox MD: **Towards a consensus regarding global signal regression for resting state functional connectivity MRI**. *NeuroImage* 2017. **154**:169–173. doi:10.1016/j.neuroimage.2016.11.052.

[29] Glasser MF, Sotiropoulos SN, Wilson JA, Coalson TS, Fischl B, Andersson JL, Xu J, Jbabdi S, Webster M, Polimeni JR *et al.*: **The minimal preprocessing pipelines for the Human Connectome Project**. *NeuroImage* 2013. **80**:105–124. doi: 10.1016/j.neuroimage.2013.04.127.

[30] Vidaurre D, Smith SM, and Woolrich MW: **Brain network dynamics are hierarchically organized in time**. *Proceedings of the National Academy of Sciences* 2017. **114**:201705120. doi:10.1073/pnas.1705120114.

[31] Ashourvan A, Pequito S, Bertolero M, Kim JZ, Bassett DS, and Litt B: **A dynamical systems framework to uncover the drivers of large-scale cortical activity**. *bioRxiv* 2019. 638718. doi:10.1101/638718.

[32] Gutierrez-Barragan D, Basson MA, Panzeri S, and Gozzi A: **Infraslow State Fluctuations Govern Spontaneous fMRI Network Dynamics**. *Current Biology* 2019. **29**:2295–2306.e5. doi:10.1016/J.CUB.2019.06.017.

[33] Chen RH, Ito T, Kulkarni KR, and Cole MW: **The human brain traverses a common activation-pattern state space across task and rest**. *Brain Connectivity* 2018. brain.2018.0586. doi:10.1089/brain.2018.0586.
