# Supplementary material for: Temporal sequences of brain activity at rest are constrained by white matter structure and modulated by cognitive demands
Source: Commun Biol. 2020 May 22;3:261. doi: 10.1038/s42003-020-0961-x (PMC7244753; doi:10.1038/s42003-020-0961-x)
Supplement: Supplementary file 4 — Reporting Summary [file 42003_2020_961_MOESM4_ESM.pdf]

nature research

Corresponding author(s): Danielle Bassett
Last updated by author(s): 2019-4-17

Reporting Summary

Nature Research wishes to improve the reproducibility of the work that we publish. This form provides structure for consistency and transparency in reporting. For further information on Nature Research policies, see Authors & Referees and the Editorial Policy Checklist.

Statistics

For all statistical analyses, confirm that the following items are present in the figure legend, table legend, main text, or Methods section.

n/a	Confirmed	
☐	☒	The exact sample size (n) for each experimental group/condition, given as a discrete number and unit of measurement
☐	☒	A statement on whether measurements were taken from distinct samples or whether the same sample was measured repeatedly
☐	☒	The statistical test(s) used AND whether they are one- or two-sided<br>*Only common tests should be described solely by name; describe more complex techniques in the Methods section.*
☐	☒	A description of all covariates tested
☐	☒	A description of any assumptions or corrections, such as tests of normality and adjustment for multiple comparisons
☐	☒	A full description of the statistical parameters including central tendency (e.g. means) or other basic estimates (e.g. regression coefficient) AND variation (e.g. standard deviation) or associated estimates of uncertainty (e.g. confidence intervals)
☐	☒	For null hypothesis testing, the test statistic (e.g. F, t, r) with confidence intervals, effect sizes, degrees of freedom and P value noted<br>*Give P values as exact values whenever suitable.*
☒	☐	For Bayesian analysis, information on the choice of priors and Markov chain Monte Carlo settings
☒	☐	For hierarchical and complex designs, identification of the appropriate level for tests and full reporting of outcomes
☐	☒	Estimates of effect sizes (e.g. Cohen's d, Pearson's r), indicating how they were calculated

Our web collection on statistics for biologists contains articles on many of the points above.

Software and code

Policy information about availability of computer code

Data collection	Details of software used in data collection can be found here: https://www.ncbi.nlm.nih.gov/pmc/articles/PMC3947233/
Data analysis	For data analysis, we used MATLAB R2017a and R2018b, as well as open-source software packages in R and Python.

For manuscripts utilizing custom algorithms or software that are central to the research but not yet described in published literature, software must be made available to editors/reviewers. We strongly encourage code deposition in a community repository (e.g. GitHub). See the Nature Research guidelines for submitting code & software for further information.

Data

Policy information about availability of data

All manuscripts must include a data availability statement. This statement should provide the following information, where applicable:

- Accession codes, unique identifiers, or web links for publicly available datasets
- A list of figures that have associated raw data
- A description of any restrictions on data availability

All structural and functional neuroimaging data are available at https://www.ncbi.nlm.nih.gov/projects/gap/cgi-bin/study.cgi?study_id=phs000607.v3.p2

Field-specific reporting

Please select the one below that is the best fit for your research. If you are not sure, read the appropriate sections before making your selection.

☒ Life sciences ☐ Behavioural & social sciences ☐ Ecological, evolutionary & environmental sciences

For a reference copy of the document with all sections, see nature.com/documents/nr-reporting-summary-flat.pdf

Life sciences study design

All studies must disclose on these points even when the disclosure is negative.

Sample size	n = 879
Data exclusions	We excluded 722 of the initial 1601 subjects for the following reasons: medical problems that may impact brain function, incidental radiologic abnormalities in brain structure, poor or incomplete FreeSurfer reconstruction of T1 images, high motion in rest or n-back fMRI scans, high signal-to-noise ratio or poor coverage in task-free or n-back task BOLD images, and failure to meet a rigorous manual and automated quality assurance protocol for DTI. Notably, our goal in constructing a sample was to compare structure-function relationships between contexts across all subjects in our sample. This analysis required highly stringent inclusion criteria that only included subjects with high quality data for rest BOLD, n-back task BOLD, and DTI.
Replication	We replicated major findings in an n=100 sample from the Human Connectome Project and in the PNC using a range of parameters relevant to our analysis.
Randomization	We compared n-back, resting state, and structural imaging between the same subjects. We did not group subjects in any way, as we aimed to study normative neurodevelopment more generally. Therefore, randomization was not relevant.
Blinding	Because there were no groupings of subjects as described above, blinding was not relevant.

Reporting for specific materials, systems and methods

We require information from authors about some types of materials, experimental systems and methods used in many studies. Here, indicate whether each material, system or method listed is relevant to your study. If you are not sure if a list item applies to your research, read the appropriate section before selecting a response.

Materials & experimental systems

n/a	Involved in the study	
☒	☐	Antibodies
☒	☐	Eukaryotic cell lines
☒	☐	Palaeontology
☒	☐	Animals and other organisms
☐	☒	Human research participants
☒	☐	Clinical data

Methods

n/a	Involved in the study	
☒	☐	ChIP-seq
☒	☐	Flow cytometry
☐	☒	MRI-based neuroimaging

Human research participants

Policy information about studies involving human research participants

Population characteristics	Resting state functional magnetic resonance imaging (fMRI), n-back task fMRI, and diffusion tensor imaging (DTI) data were obtained from n = 1601 youth who participated in a large community-based study of brain development, known as the Philadelphia Neurodevelop- mental Cohort (PNC). Here we study a sample of n = 879 participants between the ages of 8 and 22 years (mean = 15.9, s.d. = 3.3, 386 males, 493 females) with high quality diffusion imaging, rest BOLD fMRI, and n- back task BOLD fMRI data.
Recruitment	The PNC capitalized on the resources available through Center for Applied Genomics including a subject pool of approximately 50,000 genotyped youths. Critically, approximately 78% of the genotyped youths in the CAG database had provided consent to be re-contacted for future research, allowing for subjects to be approached for recruitment to the PNC. The participants were from the greater Philadelphia area and contacted after stratification by sex, age, and ethnicity.
Ethics oversight	The institutional review boards of the University of Pennsylvania and the Children's Hospital of Philadelphia approved all study procedures.

Note that full information on the approval of the study protocol must also be provided in the manuscript.

Magnetic resonance imaging

Experimental design

Design type	fractal n-back task and resting state
Design specifications	As a probe of working-memory function, we used a fractal version of the standard n-back task. The task was chosen because it has been shown to be a reliable probe of the executive system, and has the advantage of not being contaminated by lexical processing abilities that also evolve during development. The task involved presentation of complex geometric figures (fractals) for 500ms, followed by a fixed interstimulus interval of 2500ms. This occurred

under three conditions: 0-back, 1-back, and 2-back, producing different levels of WM load. In the 0-back condition, participants responded with a button press to a specified target fractal. For the 1-back condition, participants responded if the current fractal was identical to the previous one; in the 2-back condition, participants responded if the current fractal was identical to the item presented two trials previously. Each condition consisted of a 20-trial block (60 s); each level was repeated over three blocks. The target-foil ratio was 1:3 in all blocks with 45 targets and 135 foils overall. Visual instructions (9 s) preceded each block, informing the participant of the upcoming condition. The task included a total of 72s of rest while a fixation crosshair was displayed, which was distributed equally in three blocks of 24s at beginning, middle, and end of the task. Total working memory task duration was 11.6 minutes. (https://www.ncbi.nlm.nih.gov/pmc/articles/PMC3947233/)

Behavioral performance measures: We use the d-prime statistic to quantify n-back task block performance, which accounts for correct responses while discounting false positives.

Acquisition

Imaging type(s): Functional, structural, diffusion

Field strength: 3T

Sequence & imaging parameters: TR = 3000 ms; TE = 32 ms; flip angle = 90 degrees; FOV = 192 × 192 mm; matrix = 64×64; slices = 46; slice thickness = 3 mm; slice gap = 0 mm; effective voxel resolution = 3.0 × 3.0 × 3.0 mm) during the resting-state sequence and the n-back task sequence

Area of acquisition: Whole brain

Diffusion MRI: ☒ Used ☐ Not used

Parameters: For diffusion tensor imaging (DTI), 64 independent diffusion- weighted directions with a total of 7 b = 0 s/mm2 acquisi- tions were obtained over two scanning sessions to enhance reliability in structural connectivity estimates

Preprocessing

Preprocessing software: FSL, AFNI, ANTs, C3D

Normalization: template registration using MCFLIRT, boundary-based registration to individual high-resolution structural image

Normalization template: MNI305

Noise and artifact removal: De-spiking using AFNI's 3DDESPIKE utility, 36-parameter global confound regression including frame- wise motion estimates and signal from white matter and cerebrospinal fluid.

Volume censoring: We censored the first 4 volumes of each scan but did not censor high motion frames so that we could obtain an unbiased and uninterrupted estimate of temporal dynamics between individual BOLD frames. We provide additional analyses that suggest our method is minimally impacted by the inclusion of frames with greater than submillimeter framewise displacement.

Statistical modeling & inference

Model type and settings: t-tests, non-parametric permutation tests, comparing distributions of null models to observed data non-parametrically, multiple linear regression.

Effect(s) tested: We compare summary metrics of brain dynamics between resting state and task, and relate those metrics to structural connectivity, age, and cognitive performance.

Specify type of analysis: ☒ Whole brain ☐ ROI-based ☐ Both

Statistic type for inference (See Eklund et al. 2016): We use k-means clustering to assign each BOLD volume to clusters, then compare summary metrics of the dynamics of those clusters (i.e. transition probabilities between and dwell times in each cluster) between resting state and n-back task and relate those metrics to age, cognitive performance, and structural connectivity. We do not use SPM or other common packages for fMRI analysis.

Correction: We applied a Bonferroni correction over all statistical tests for each analytic question.

Models & analysis

n/a	Involved in the study	
☒	☐	Functional and/or effective connectivity
☒	☐	Graph analysis
☐	☒	Multivariate modeling or predictive analysis

Multivariate modeling and predictive analysis: We use k-means clustering to group BOLD frames from resting state and n-back task for all subjects, and

Multivariate modeling and predictive analysis

then relate summary metrics of the temporal dynamics of those clusters to age, structural connectivity, and cognitive performance.
